# Small-Molecule Inhibition of Rho/MKL/SRF Transcription in Prostate Cancer Cells: Modulation of Cell Cycle, ER Stress, and Metastasis Gene Networks

**DOI:** 10.3390/microarrays5020013

**Published:** 2016-05-28

**Authors:** Chris R. Evelyn, Erika M. Lisabeth, Susan M. Wade, Andrew J. Haak, Craig N. Johnson, Elizabeth R. Lawlor, Richard R. Neubig

**Affiliations:** 1 Department of Pharmacology, University of Michigan, Ann Arbor, MI 48109, USA; Chris.Evelyn@cchmc.org (C.R.E.); suemwade@umich.edu (S.M.W.); Haak.Andrew@mayo.edu (A.J.H.); 2 Department of Pharmacology & Toxicology, Michigan State University, East Lansing, MI 48824, USA; matheser@msu.edu; 3 University of Michigan Microarray Core, University of Michigan Comprehensive Cancer Center, Ann Arbor, MI 48109, USA; johnscrn@umich.edu; 4 Departments of Pediatrics and Pathology, University of Michigan, Ann Arbor, MI 48109, USA; elawlor@umich.edu

**Keywords:** metastasis, transcription, cell cycle, Rho

## Abstract

Metastasis is the major cause of cancer deaths and control of gene transcription has emerged as a critical contributing factor. RhoA- and RhoC-induced gene transcription via the actin-regulated transcriptional co-activator megakaryocytic leukemia (MKL) and serum response factor (SRF) drive metastasis in breast cancer and melanoma. We recently identified a compound, CCG-1423, which blocks Rho/MKL/SRF-mediated transcription and inhibits PC-3 prostate cancer cell invasion. Here, we undertook a genome-wide expression study in PC-3 cells to explore the mechanism and function of this compound. There was significant overlap in the genes modulated by CCG-1423 and Latrunculin B (Lat B), which blocks the Rho/MKL/SRF pathway by preventing actin polymerization. In contrast, the general transcription inhibitor 5,6-dichloro-1-β-d-ribofuranosyl-1H-benzimidazole (DRB) showed a markedly different pattern. Effects of CCG-1423 and Lat B on gene expression correlated with literature studies of MKL knock-down. Gene sets involved in DNA synthesis and repair, G1/S transition, and apoptosis were modulated by CCG-1423. It also upregulated genes involved in endoplasmic reticulum stress. Targets of the known Rho target transcription factor family E2F and genes related to melanoma progression and metastasis were strongly suppressed by CCG-1423. These results confirm the ability of our compound to inhibit expression of numerous Rho/MKL-dependent genes and show effects on stress pathways as well. This suggests a novel approach to targeting aggressive cancers and metastasis.

## 1. Introduction

Metastasis is a complex multi-step process that enables tumor cells to disseminate from their site of origin to colonize distant tissue and organ sites [1,2,3,4,5]. Recent evidence has identified alterations in gene expression related to metastasis with several classes of genes implicated [4]. Furthermore, numerous gene signatures have been identified that can predict the aggressiveness and metastatic potential of numerous cancers [6,7].

Metastasis initiation genes enable tumor cells to invade the surrounding tissue, disseminate, and intravasate [4]. This includes genes related to epithelial-mesenchymal transition (EMT, e.g., Twist1, Snai1, and Snai2, [8]) as well as hepatocyte growth factor (HGF) pathway-dependent genes [9]. Genes that enable tumor cells to extravasate and survive at the secondary tissue site contribute to metastasis progression including prostaglandin G/H synthase 2 (PTGS2), epiregulin, and angiopoietin-like 4 (ANGPTL4) [4,10,11]. Lastly, metastasis virulence genes, such as parathyroid hormone-related protein (PTHRP) and interleukin 11 (*IL11*) enable tumor cells to colonize and survive at the distant secondary tumor site [4,12,13,14]. Therefore, gene expression plays a vital role in the complex process of cancer metastasis.

The small GTPases RhoA and RhoC are strongly implicated in metastasis [15,16]. While most attention has been focused on the cytoskeletal actions of RhoA and RhoC [16], they also produce transcriptional effects [17,18,19,20]. This is mediated through the actin-dependent translocation of the transcriptional co-activator MKL (MAL, MTRF, or BSAC) to the nucleus where MKL binds with serum response factor (SRF) at serum response elements (SRE). Transcriptional targets include c-Fos, matrix metalloproteinases, genes related to EMT [21], and genes responsive to E2F, c-Myc, and c-Jun transcription factors [18]. Recently, MKL and SRF were shown to play a key role in metastasis in melanoma and breast cancer xenograft systems [22] providing a strong link to the transcriptional effects of RhoA or RhoC in these processes.

In addition, we identified CCG-1423, a nM-potency small-molecule inhibitor of RhoA/C transcriptional signaling [23]. This compound inhibits SRE promoter activation by RhoA and -C and MKL in both HEK-293T cells and in PC-3 prostate cancer cells. It also inhibits Matrigel^®^ invasion by PC-3 cells but does not inhibit invasion by the Gαi/ras-dependent SKOV-3 ovarian cancer cells [23]. This supports the role for RhoC in invasion by PC-3 cells [24] and is consistent with the recent demonstration of a role for MKL [22] implicating transcriptional outputs from the Rho pathway in invasion by certain aggressive cancers.

To gain further insight into the mechanism of action of CCG-1423, we undertook a genome-wide gene expression analysis of its effects on PC-3 prostate cancer cells utilizing conditions employed in our PC-3 cell Matrigel invasion assays. There were strong overlaps between genes regulated by CCG-1423 and by Lat B, which is known to disrupt Rho-mediated gene transcription. Furthermore, targets of the Rho-regulated transcription factor E2F related to G1/S transition were strongly suppressed by CCG-1423. Finally, there was a clear correlation between CCG-1423-regulated genes and those modulated by RNAi-mediated suppression of the transcriptional co-activator MKL—especially in melanoma where Rho/MKL function is strongly implicated in metastasis. Beyond effects on the Rho/MKL pathways, CCG-1423 also stimulated expression of stress-responsive genes such as activated transcription factors 3 and 4 (ATF3, ATF4) and DNA-damage-induced transcript 3 (DDIT3, also referred to as CHOP) suggesting that alternative mechanisms contribute to the actions of this compound.

## 2. Materials and Methods

### 2.1. Cell Lines and Reagents

Dimethyl Sulfoxide (DMSO), the marine toxin Latrunculin B (Lat B), and the RNA Polymerase carboxyl-terminal domain kinases inhibitor DRB (5,6-Dichlorobenzimidazole 1-β-d-ribofuranoside) were all purchased from Sigma (St. Louis, MO, USA). The RhoA/C transcriptional pathway inhibitor, CCG-1423 (*N*-(2-(4-chloroanilino)-1-methyl-2-oxoethoxy)-3,5-bis(trifluoromethyl) benzamide), was from Cayman Chemical (Ann Arbor, MI, USA). The PC-3 cell line was a kind gift from Kenneth Pienta (University of Michigan, Ann Arbor, MI, USA). The RNAqueous^®^ RNA isolation kit was purchased from Ambion (Austin, TX, USA). The TURBO DNAse-free kit was purchased from Ambion (Austin). The TaqMan^®^ Reverse-Transcription Reagents Kit was purchased from Applied Biosystems (Foster City, CA, USA). The Brilliant^®^ II SYBR^®^ Green QPCR Master Mix with low Rox dye was purchased from Stratagene (La Jolla, CA, USA).

### 2.2. Cell Culture

The PC-3 prostate cancer epithelial cell line was normally maintained in DMEM (Invitrogen, Carlsbad, CA, USA, cat.#: 11995) containing 10% fetal bovine serum (FBS), 100 units/mL penicillin, and 100 µg/mL streptomycin at 37 °C in 5% CO_2_.

### 2.3. Microarray Analysis

PC-3 cells (9 × 10^5^) were seeded into individual 60 mm dishes. Twenty-four hours later, they were serum-starved (0.5% FBS) for twenty-four hours. Cells were then treated for an additional two or twenty-four hours with DMSO (1.0%), 3 µM of CCG-1423, 0.5 µM of Lat B, or 50 µM of DRB under serum-starved conditions (0.5% FBS). Cells from three dishes for each condition were lysed and RNA was isolated using the RNAqueous^®^ kit from Ambion following the manufacturer’s directions. cDNA and biotin-labeled cRNA synthesis, hybridization to the human U133 plus 2.0 Affymetrix gene chips, scanning of the gene chips, and analysis of the data images were all performed at the University of Michigan Comprehensive Cancer Center (UMCCC) Affymetrix and Microarray Core Facility in Ann Arbor, MI, USA. Expression values for each probeset on the chip were calculated using a robust multi-array average (RMA) [25] and were expressed as log2 transformed data. Fold-change values for each probeset were calculated by subtracting the average log2 (expression) values for the compound samples from the average log2 (expression) values of the DMSO samples.

**Figure 1:**
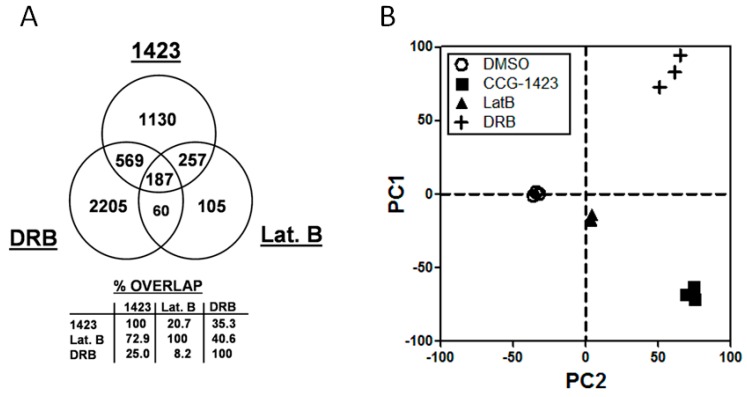
Microarray analysis of regulated genes at 24 h. PC-3 cells were treated with 3 µM of CCG-1423, 0.5 µM of Lat B, and 50 µM of DRB under serum-starved conditions (0.5% FBS) for 24 h and gene expression assessed with an Affymetrix gene chip. (**A**) The Venn diagram shows the number of genes regulated (stimulated or inhibited) by CCG-1423, Lat B, and DRB with ≥2-fold change. The percentages of genes coordinately regulated by CCG-1423 and Lat B, or Lat B, and DRB are indicated; (**B**) A principal components analysis of the differentially regulated genes shows distinct patterns for Lat B and CCG-1423 *vs.* DRB.

For simple comparisons of different treatment results and preliminary candidate selection, a record for each probeset showing at least a two-fold change (*i.e.*, log2-fold change >1 for a decrease or <−1 for an increase in expression) was imported into a MySQL^®^ database (Sun Microsystems, Santa Clara, CA, USA). Literature microarray datasets [20,22] were also imported for comparisons. Queries of those datasets were utilized to obtain counts for genes with altered gene expression, identify overlaps between datasets, identify highly regulated genes (log2-fold change >3 or <−3), filter by gene ontology (GO) categories to generate Table 1 and Table 2 and the Venn diagram in Figure 1.

### 2.4. Bioinformatic Analysis

Gene set enrichment analysis (GSEA) was also done to identify both KEGG categories and literature gene expression datasets that were selectively modulated by our compound [26]. Gene sets were curated and organized in the Broad Institutes Molecular Signature Database version 3.0. GSEA was performed using the R statistical environment and the PGSEA Bioconductor package. For each gene set a Z-score was created testing whether the mean fold changes (genes of gene set/average of controls) were statistically different from the mean fold changes of the entire microarray data set. Linear models were then used on to find gene sets with statistical different Z-scores between the two experimental groups.

Finally, to assess enrichment of specific GO categories using an alternate method, genes that were significantly induced or repressed by CCG-1423 with log2-fold changes of greater than ±1 (absolute fold-change >2) were uploaded for analysis using DAVID bioinformatics resources [27]. GO biologic process, molecular function and cell component category representation were determined for both gene lists and GO categories with Benjamini corrected *p*-values (false discovery rate, FDR) of <0.05 were considered to be significantly over-represented.

### 2.5. Quantitative Real-Time Polymerase Chain Reaction (qRT-PCR)

To confirm mRNA changes from the microarray results for a set of candidate genes, PC-3 cells (9 × 10^5^) were seeded into 60 mm dishes. Twenty-four hours after plating, PC-3 cells were serum-starved (0.5% FBS). Cells were then treated for an additional twenty-four hours with DMSO (0.03% or 1.0%) or compound under serum-starved conditions (0.5% FBS) for PC-3 cells. Cells were lysed and RNA was isolated using the RNAqueous^®^ kit from Ambion following the manufacturer’s directions. RNA (10 µg) was treated with DNAse using the TURBO DNA-free kit from Ambion following the manufacturer’s directions. DNAse-treated RNA (1 µg) was used as a template for synthesizing cDNA utilizing the Taqman^®^ Reverse-Transcription Reagents kit. The components used for the reverse-transcription reaction were 1× reverse transcription buffer, 5.6 mM MgCl_2_, 2 mM dNTPs, oligo dT, RNAse inhibitor, reverse transcriptase enzyme, sterile water, and 1 µg of RNA. The sequential order of reaction conditions was: (i) 25 °C for 10 min; (ii) 48 °C for 30 min; and (iii) 95 °C for 5 min and carried out in a Biometra TGradient thermocycler (Analytikijena, Gottingen, Germany). The qRT-PCR reaction was performed using 5 µL of cDNA sample per well in a 25 µL final reaction volume in a 96-well qPCR plate, and the Brilliant^®^ II SYBR^®^ Green QPCR Master Mix with low Rox dye from Stratagene in a Stratagene MX3000P^®^ QPCR System. The reaction mix consisted of 1× of the Brilliant^®^ II SYBR^®^ Green QPCR Master Mix with low Rox dye, 200 nM each of both forward and reverse primers, and sterile water. The sequential order of reaction conditions was: (i) 95 °C for 10 min; (ii) 95 °C for 30 s; (iii) 55 °C for 1 min; and (iv) 72 °C for 30 s. Steps 2 through 4 were carried out for 50 cycles. The primer sequences used were *GAPDH*: Fwd-5′-GGAAGGACTCATGACCACAG-3′, Rev-5′-ACAGTCTTCTGGGTGGCAGTGATG-3′ (base-pairs were corrected to match human sequence) [20]; *RGS4*: Fwd-5′-TTCCCACAACAAGAAGGACAAAG-3′, Rev-5′-TGATTCAGCCCATTTCTTGAC-3′ (base-pairs were corrected to match human sequence) [28]; *RGS7*: Fwd-5′-CCTTCTAACCCATGGCTGTC-3′, Rev-5′-TTTTTCAGGTCCTCCACTGC-3′ [29]; *CTGF*: Fwd-5′-CAGAGTGGAGCGCCTGTTC-3′, Rev-5′-CTGCAGGAGGCGTTGTCAT-3′ [30]; *SOX9*: Fwd-5′-CAACCAGAATTCCCTTTGGA-3′, Rev-5′-TGCTCCATTTAGCCAAGGTT-3′ [31]; *ATF3*: Fwd-5′- TCAAGGAAGAGCTGAGGTTTGCCA-3′, Rev-5′-CTTCTTGTTTCGGCACTTTGAGC-3′; *ATF4*: Fwd-5′-CTGCTGAATGCCGTGAGAA-3′, Rev-5′-GCGTATTAGGGGCAGCAGT-3′; *DDIT3 (CHOP)*: Fwd-5′-CCATCTCTGCAGTTGGATCA-3′, Rev-5′-CCAAAATCAGAGCTGGAACC-3′. *GAPDH* gene expression was utilized as an internal control. The relative mRNA gene expression was calculated using the following formula: 2^−(∆*C*t)^ where the ∆*C*_t_ value = *C*_t_ value of Sample—*C*_t_ value of GAPDH. Fold changes were calculated by dividing the 2^−(∆*C*t)^ of the compound sample by the 2^−(∆*C*t)^ of the DMSO control sample.

## 3. Results

### 3.1. Microarray Analysis

For our microarray analysis, we chose to use unstimulated PC-3 cells (grown in 0.5% serum) to mimic the conditions in which functional inhibition of PC-3 cell matrix invasion was demonstrated [23]. We also utilized the actin cytoskeleton inhibitor, Lat B, as a positive control compound that inhibits the RhoA/C/MKL/SRF transcriptional pathway via disruption of actin polymerization and the transcription elongation inhibitor, DRB, as a “non-specific” control to inhibit general RNA Polymerase II-mediated transcription.

After two hours of compound treatment, we observed only a few transcripts with >2-fold changes (Table 1). Interestingly, both CCG-1423 and Lat B had primarily stimulatory effects on gene expression. CCG-1423 increased levels of 48 transcripts out of 49 total transcripts changed, while Lat B increased expression of 15 genes out of 18 total changed gene transcripts (Table 1 and Table S1). In contrast, the transcription inhibition control compound, DRB, had a marked effect in the opposite direction at 2 h, primarily reducing mRNA levels (714 gene transcripts showed decreased levels out of 719 total gene transcripts changed—Table 1). Of the 15 genes up-regulated by Lat B at 2 h, five of them were also up-regulated by CCG-1423 (JUN, ERRFI1, FBXO32, GDF15, and GEM). This is consistent with the depletion of MKL in invasive breast cancer and melanoma cell lines, where the authors also observed an increase in *Jun* and *FBXO32* RNA levels [22]. In addition, CCG-1423 induced the stress-responsive genes *ATF3* and *DDIT3* (also referred to as CHOP) while Lat B did not.

However, at 24 h, a large number of genes had their expression altered by the three compounds by ≥2 fold (Table 1 and Figure 1). Not surprisingly, DRB modulated expression of the greatest number of genes (3020), while CCG-1423 also affected a substantial number (2142). Lat B had the most selective effect, modulating expression of only 608 genes. Unlike the 2 h time point, at 24 h, all three compounds downregulated 1.5–2 times more transcripts than they up-regulated (Table 1). There is a clear relation between the genes modulated by CCG-1423 and by Lat B (Figure 1): 73% of genes modulated by Lat B were also altered by CCG-1423. Furthermore, 427 of the 444 genes regulated by both Lat B and CCG-1423 changed in the same direction (Table S1). While DRB modulated almost 50% more genes than did CCG-1423, DRB only shared 247 modulated genes in common with Lat B *vs.* 444 for CCG-1423. The similarity of the effects of CCG-1423 and Lat B is confirmed in a principal components analysis (Figure 1B). The data points for Lat B lie essentially on the line connecting the DMSO control and the CCG-1423 samples, consistent with a high proportion of shared genes but the more limited effect of Lat B. In contrast, the DRB samples are clearly in a different sector of the principal components graph.

It is not surprising that CCG-1423 and Lat B have strongly overlapping transcriptional effects since CCG-1423 was initially discovered in a screen for inhibition of a RhoA/C-responsive, MKL/SRF dependent luciferase reporter (SRE.L) [23]. Indeed, Lat B was used as a positive control for the screen due to its ability to block RhoA/C-SRE-mediated gene transcription by preventing actin polymerization. Although CCG-1423 does not block the RhoA/C transcription pathway at the level of actin polymerization [23], it clearly does modulate the same set of genes as does Lat B (although it also targets a broader array).

To further assess whether CCG-1423 could modulate the same target genes as reported for MKL-1, we compared our gene expression data with results from two studies in the literature documenting MKL-dependent gene expression. Prywes and colleagues tested the effect of overexpressing a dominant-negative MKL1 protein on serum-induced gene expression in NIH-3T3 cells [20]. Treisman and colleagues tested the effect of MKL1/2 (also referred to as MRTF A/B) shRNA knockdown on global gene expression in invasive breast cancer (MDA-MB-231) and melanoma (B16F2) cell lines [22]. CCG-1423 regulated 18%–26% of MKL-dependent genes (Table 2). Surprisingly, Lat B only regulated a very small percentage of the MKL-dependent genes from those three studies (0%–17%) while DRB regulated a similar percentage of genes as did CCG-1423 (18%–25%, Table 2). Since the overlap between MKL-1 dependent genes and the genes regulated by CCG-1423 is relatively low, this suggests that CCG-1423 may affect a broader transcriptional mechanism.

**Figure 2:**
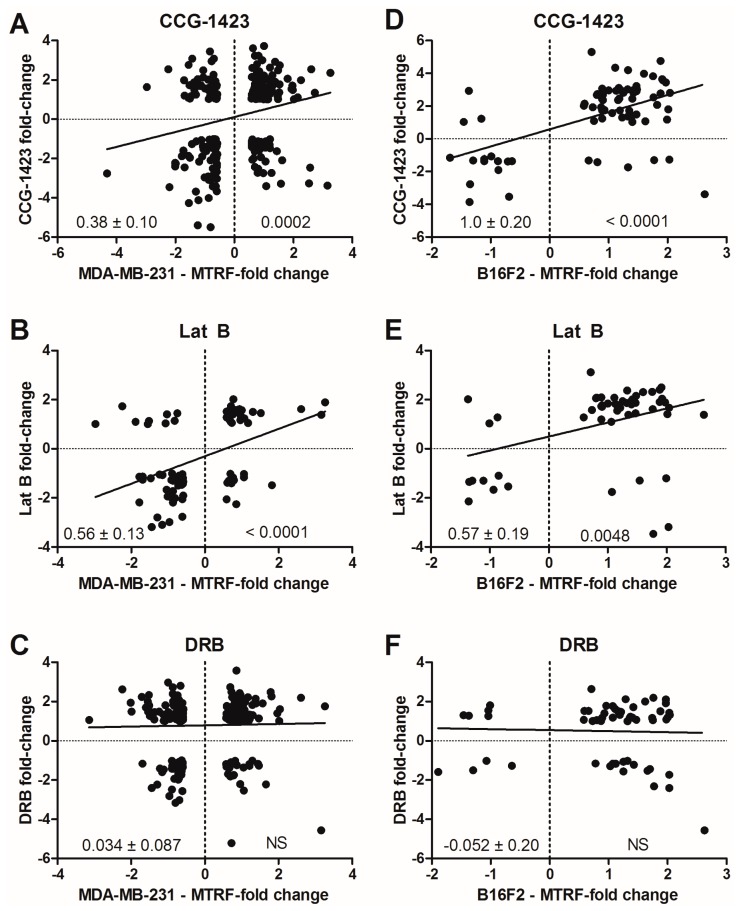
Magnitude of expression changes observed with compounds compared to effects of MKL/MTRF suppression. Gene sets and the fold-change (log2) were compiled from our microarray data sets and from the data of Medjkane *et al.* [22]. For the overlapping genes, the magnitude of the change by CCG-1423 is plotted relative to the magnitude of the change induced by combined shRNA suppression of MRTF-A and MRTF-B (also referred to as MKL-1 and MKL-2). Linear regression analysis was performed to provide an indication of the extent to which the trends were similar for the two different interventions. Numbers in lower left quadrant are mean ± SD of slope and that in the lower right quadrant is the *p* value when significant. (**A**–**C**) Comparison of compound addition on gene expression to the fold change observed in MDA-MB-231 breast carcinoma cells with shRNA against MRTF-A and MRTF-B; (**D**–**F**) Same analysis but compared to B16F2 melanoma cells with shRNA against MRTF-A and MRTF-B.

Although CCG-1423 did not affect any more MKL-regulated genes than did DRB, the direction of effects by CCG-1423 on the two cancer cell lines correlated with that of MKL knockdown (Figure 2A,D). In contrast, the effects of DRB showed no directionality (Figure 2C,F). Furthermore, even the few MKL-dependent genes affected by Lat B showed the same directionality and a significant correlation in magnitude with the effects of MKL knock-down (Figure 2B,E). Both CCG-1423 and Lat B showed more consistent effects on the melanoma cell line (Table 2 and Figure 2) than they did on the breast cancer cells, which is in line with our prior data on the strong biological effect of CCG-1423 on invasive melanoma [23]. The CCG-1423 modulated genes were also enriched in those predicting aggressive behavior of clinical melanomas (see below). Although CCG-1423 clearly modulates a broader set of genes than does the genetic suppression of MKL function, our data do support an effect on MKL-dependent genes—most significantly those identified in the B16F2 melanoma system.

### 3.2. Candidate Gene Analysis

To help focus our analysis of the large number of modulated genes, we used several criteria to identify potential genes of interest with respect to the ability of CCG-1423 to block PC-3 cell matrix invasion. Utilizing metastasis-related gene ontology (GO) categories as a filter, we identified 203 gene transcripts (Table S2) out of the 2142 total gene transcripts that are regulated by CCG-1423 at 24 h. To further narrow the candidate genes to follow-up upon, we chose those where CCG-1423 caused at least an eight-fold change in gene expression leaving us with 16 candidate gene transcripts. Of those, we chose four that the literature provided evidence related to invasion or metastasis. These included regulator of G-protein signaling 4 (RGS4) and 7 (RGS7), which were both upregulated by CCG-1423, and CTGF (connective tissue growth factor), and SOX9 (sex determining region Y-box 9, also referred to as campomelic dysplasia, autosomal sex reversal) which were down-regulated.

Several studies have shown a relationship between RGS protein expression and cancer invasion. RGS4 was previously found to be down-regulated in invasive cancers compared to either non-invasive cancer or normal epithelial cells for both breast and ovarian cancer systems [32,33]. In NIH3T3 mouse fibroblast cells, constitutively activated Gαo-subunit stimulated colony formation in a Stat3-dependent manner [34]. RGS7-mediated suppression of Gαo signaling could reduce cancer progression dependent upon Gαo signaling pathways. Interestingly, RGS4 has several predicted SRF binding sites located within its promoter [35], suggesting that modulation of RGS4 mRNA levels by CCG-1423 may be through a SRF-dependent mechanism.

CTGF and SOX9, both of which were found to be downregulated in this study, have also been implicated in cancer invasion. Expression of CTGF is known to be induced by RhoA signaling and it is over-expressed in many types of cancers, including head and neck squamous cell carcinoma [36], pancreatic cancer [37], gastric cancer [38], and in pre-B acute lymphoblastic leukaemia [39]. In gastric cancer, CTGF overexpression correlated with poor patient survival [38] and in pancreatic cancer CTGF stimulated tumor growth *in vitro* and *in vivo* [37]. SOX9 is overexpressed in both colorectal cancer [40] and hormone-refractory prostate cancer [41] and has been correlated with poor patient survival in colorectal cancer [40] and enhanced *in vivo* tumor growth, angiogenesis, and invasion in a LNCaP prostate cancer xenograft model [42]. Therefore, inhibition of CTGF and/or SOX9 expression by CCG-1423 could contribute to reduced PC-3 cell invasion.

In order to confirm the microarray results, we tested by qRT-PCR the effect of CCG-1423 on levels of *RGS4*, *RGS7*, *CTGF*, and *SOX9* mRNA in PC-3 prostate cancer cells. As expected, *CCG-1423* increased mRNA levels of *RGS4* and *RGS7* and decreased *CTGF* and *SOX9* gene expression (Table 3). Overall, the magnitudes of CCG-1423-induced changes were similar by qRT-PCR and microarray quantification for all four candidate genes.

### 3.3. Time-Course of CCG-1423 Regulated Gene Expression

**Figure 3:**
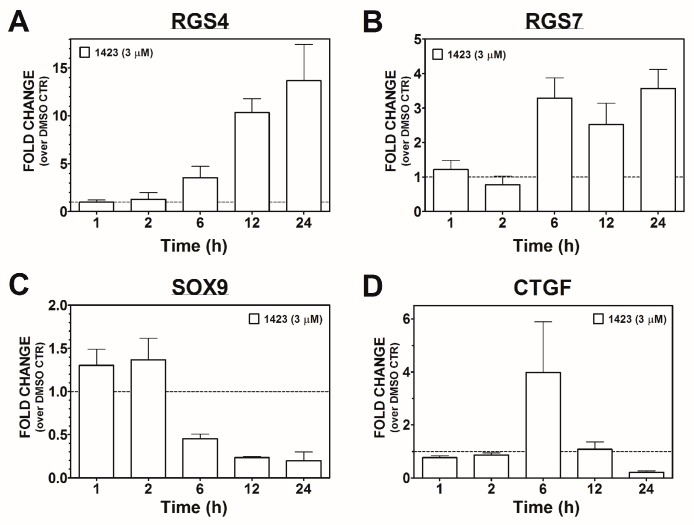
Time-course of CCG-1423 effect on PC-3 cell gene expression. PC-3 cells were treated with 3 µM of CCG-1423 under serum-starved (0.5% FBS) conditions for 1, 2, 6, 12, and 24 h. Four genes: *RGS4* (**A**); *RGS7* (**B**); *SOX9* (**C**); and *CTGF* (**D**), identified as metastasis candidates, were tested using qRT-PCR as described in the Materials and Methods. (**A**–**D**) Data represent mean ± SEM fold-change values (over DMSO Control) for three separate experiments.

In order to better understand the timing of CCG-1423-regulated gene expression, we studied the effect of *CCG-1423* on *RGS4*, *RGS7*, *CTGF*, and *SOX9* gene expression in PC-3 prostate cancer cells from 1 to 24 h (Figure 3). *RGS4* gene expression increased in a time-dependent manner peaking at 12–24 h while *RGS7* gene expression reached its maximum by 6 h (Figure 3B). Interestingly, CCG-1423 stimulated *CTGF* gene expression at six hours but it subsequently decreased at 12 and 24 h (Figure 3D) to produce the strong reduction observed in the microarray data. Finally, CCG-1423 inhibited *SOX9* gene expression reaching peak suppression by 12–24 h (Figure 3C). Consequently it takes between 6 h (*RGS7*) and 24 h (*RGS4* and *CTGF*) of CCG-1423 treatment to obtain maximal effects on expression of these candidate mRNAs.

### 3.4. Global Analysis

**Figure 4:**
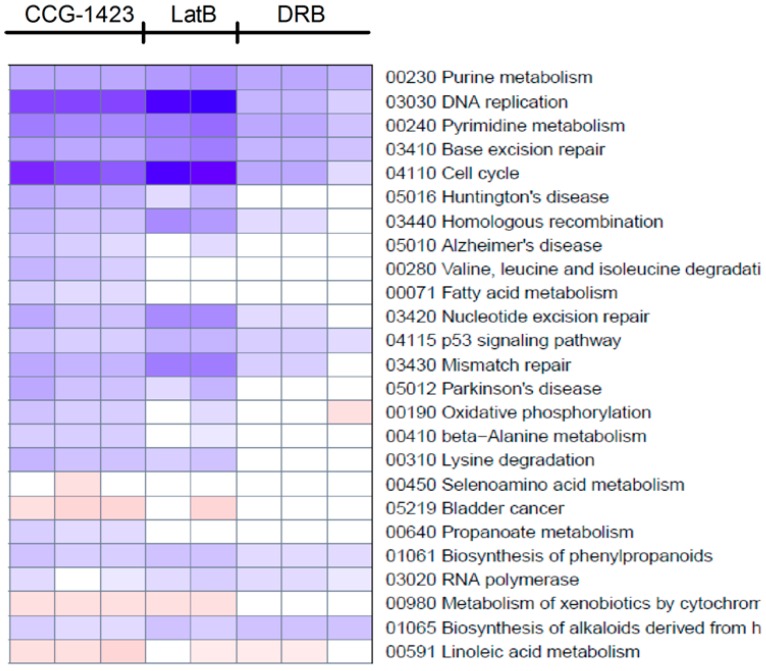
Concept map of gene expression changes in PC-3 cells induced by three treatments. KEGG pathways, defined by MSigDB (Broad Institute version 3.0), were examined using Gene set enrichment analysis (GSEA) and the entire microarray data set. The top 25 KEGG pathways significantly associated with changes induced by CCG-1423 (3 µM) are shown along with the related changes induced by Lat B (0.5 µM) and DRB (30 µM). All three compounds produced significant effects on genes involved in purine and pyrimidine metabolism while CCG-1423 and Lat B had more selective effects on cell cycle and DNA replication. Colors represent *Z*-scores with blue indicating that genes of the set were downregulated and red indicating that genes of the set were up-regulated. The darkness represents the level of significance. White means that the adjusted *p* value is >0.05. One of the 24-h Lat B samples was lost during processing for the microarray so there are only two replicates.

In addition to the candidate gene approach, we undertook three global analyses of gene expression to assess the possibility of actions at the level of gene networks. This would not be surprising, in light of complex gene programs induced by RhoA family members in NIH-3T3 cells [18]. Comparisons of the effects of CCG-1423 and the control compounds (Lat B and DRB) were done for functionally defined gene sets (KEGG and GO pathways) and literature microarray datasets (Broad MSigDB version 3.0). Genes modulated by CCG-1423 in PC-3 cells showed a significant correlation with 30 KEGG pathway sets (adjusted *p* value < 0.05). Similarly, Lat B and DRB showed significant correlations with 21 and 34 categories, respectively. A heat-map for the top 25 KEGG gene categories regulated by CCG-1423 is shown in Figure 4. All three compounds (CCG-1423, Lat B, and DRB) produced highly significant effects on genes involved in purine and pyrimidine metabolism and DNA repair. The heat map for Lat B is very similar to that for CCG-1423, and both had marked effects on genes involved in cell cycle and DNA replication. (Figure S1A). Indeed, all 10 of the top categories for Lat B were within the top 15 for CCG-1423. In contrast, the top 25 gene categories regulated by DRB were quite different (Figure S1B) and only five of the top 10 gene categories modulated by DRB are within even the top 25 for CCG-1423. The concordance between effects of CCG-1423 and Lat B on KEGG pathway genes, and specifically on cell cycle and DNA replication, is consistent with the principal components analysis in Figure 1C. In addition, treatment of LPA stimulated PC3 cells with CCG-1423 reduced DNA replication, which further validates our microarray data [23].

**Figure 5:**
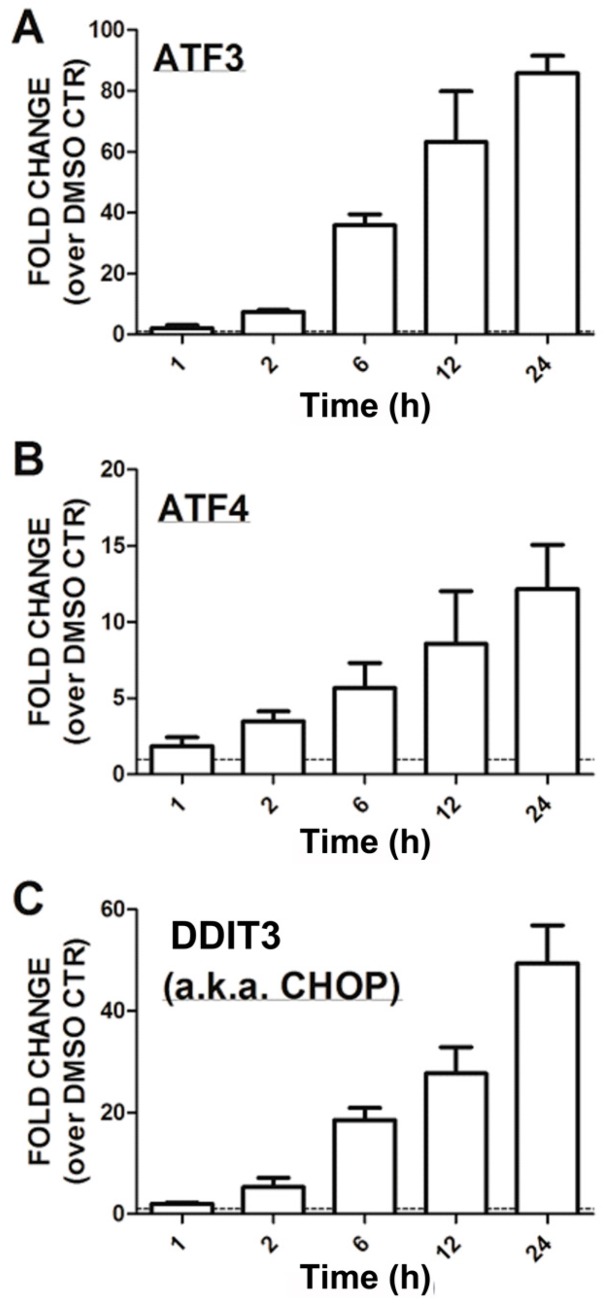
qRT-PCR confirmation of ER Stress-related gene expression. PC-3 cells were treated with 3 µM CCG-1423 for the indicated times. RNA was isolated and qRT-PCR performed as described. Expression of (**A**) ATF3, (**B**) ATF4, and (**C**) CHOP (also referred to as DDIT3) is expressed as the fold change compared to the DMSO control samples (24-h with DMSO). All expression levels were normalized relative to the expression of GAPDH.

We also assessed the most strongly modulated genes according to GO category (biological process, molecular function, and cellular compartment) using David [43]. This analysis separately assesses genes that are up-regulated and those that are down-regulated. The biological processes that showed the greatest effects (Table S3, false discovery rate, FDR < 10^−8^, red font) were all down-regulated and were related to cell cycle and DNA replication and repair. This further validates the results from the GSEA/KEGG analysis. With cell cycle, DNA replication, and DNA repair as the biological process, the molecular functions with lowest FDR, as expected, were related to nucleotide/nucleoside binding and the cellular compartments were chromosome, nucleus, and mitotic spindle. The most highly significant up-regulated genes (FDR < 10^−5^) were related to apoptosis followed closely by endoplasmic reticulum stress and unfolded protein response (FDR < 10^−4^). Markers of the unfolded protein response (ATF3, ATF4, and DDIT3—also referred to as CHOP) were retested by qRT-PCR and their mRNA levels were markedly increased at 24 h (10–80-fold, Figure 5).

The literature datasets in MSiGDB (GSEA, Broad Institute, version 3.0) also revealed important similarities with the effects of CCG-1423 on gene expression. Of the 50 related gene datasets (Figure S2), 36 were not significantly affected by DRB (Table S4) so we consider them more related to the CCG-1423 mechanism. Of those, 25 were shared with Lat B further confirming the close similarity of the effects of CCG-1423 and Lat B. In this analysis, we will focus on those gene sets (36/50) that were not also associated with the effects of the transcription elongation inhibitor DRB. As would be predicted from the KEGG and GO analysis, the greatest number of related sets had to do with proliferation, DNA synthesis, and cell cycle. Several other commonalities were also apparent in those gene sets specific for CCG-1423 but not DRB.

For example, genes that are targets of the transcription factor family E2F were strongly downregulated (marked blue in Table S4). Specifically DHFR, TK1, and CCNE2 (cyclin E2) were all downregulated either ~2-fold (DHFR) or ~8-fold (TK1 and CCNE2) by treatment with CCG-1423 and ~2-fold (DHFR) or ~4-fold (TK1 and CCNE2) by Lat B. Interestingly, E2F-regulated transcription has been shown to be one of three key gene programs (*E2F*, *Myc*, and *Jun*) induced by RhoA or RhoC in NIH-3T3 cells [18], [23]. The ability of CCG-1423 to shut down Rho-regulated gene transcription, including suppression of the E2F transcription pathway, is unique and may represent an important new tool for disrupting Rho-mediated transformation and invasion.

The effect of our compound also had significant overlap with gene sets found in metastatic cancers. Two gene sets of particular interest relate to melanoma since CCG-1423 was found to be most potent at inhibiting the highly metastatic variant A375M2 line [23]. WINNEPENNINCKX_MELANOMA_METASTASIS_UP and KAUFFMANN_MELANOMA_ RELAPSE_UP gene sets (marked red in Table S4) were both strongly downregulated by CCG-1423 in PC-3 cells. Strong downregulation of genes associated with metastasis and relapse could provide an important approach to preventing those processes and/or killing cells engaging in those processes. Another very highly associated gene set (marked yellow in Table S4) includes those that are upregulated in human gastric cancer cells after selection for resistance to doxorubicin (KANG_DOXORUBICIN_RESISTANCE_UP). Genes upregulated by doxorubicin resistance but downregulated by CCG-1423 include TOP2A and RRM1. This suggests the potential for synergy between doxorubicin and our compound. Finally, two gene sets (also marked yellow in Table S4) that were strongly up-regulated by CCG-1423 relate to effects of agents that represent novel potential cancer therapies.

## 4. Discussion

RhoA and RhoC have been strongly implicated in the aggressiveness and metastasis of a number of cancer types, including breast, prostate, and melanoma [15,16,44], but the precise mechanisms underlying those effects are not clear. Furthermore, there are no effective targeted therapies to combat Rho-mediated functions. Recent evidence [22] points to a critical role for gene transcriptional mechanisms downstream of Rho (*i.e.*, MKL and SRF) and we have identified a series of small molecule compounds, including CCG-1423, that block Rho/MKL-stimulated gene transcription [23,45]. In the present study, we used mRNA microarray analysis in the RhoC-dependent aggressive PC-3 human prostate cancer cell line [24,46] to show that CCG-1423 does effectively perturb Rho-regulated transcription pathways. While no single gene was identified whose modulation explains the effects of our compounds, we demonstrate a strong connection to E2F family transcription factor-regulated genes. This is consistent with data showing that RhoA and RhoC-mediated transformation of NIH 3T3 cells is dependent on activation of E2F transcriptional mechanisms [18].

This study identifies other biological mechanisms (cell cycle, DNA replication and repair, G1/S checkpoint, apoptosis, and ER stress) influenced by our novel Rho transcription pathway inhibitor, CCG-1423, in PC-3 prostate cancer cells. It is clear that this compound does not produce a broad or general inhibition of transcription since its effect is quite distinct from that of the transcription elongation inhibitor DRB. However, the number of genes modulated by CCG-1423 is significantly greater than those affected by actin cytoskeletal block by Lat B (this report) as well as those modulated by the ROCK inhibitor Y-27632 in NIH 3T3 cells [18]. This is consistent with the concept that Rho activation can engage multiple transcription programs but only a subset are blocked by existing targeted Rho pathway inhibitors [18]. Alternatively, the molecular mechanism of CCG-1423 may involve a more general cellular target than just Rho or its downstream transcription coactivator MKL. We have identified several transcriptional programs that are modulated in PC-3 human prostate cancer cells by our Rho/MKL/SRF transcription inhibitor CCG-1423. In particular the strong suppression of genes responsive to the E2F family of transcription factors is of great interest. E2F plays a key role in the G1/S checkpoint resulting in increased DNA synthesis and cancer cell proliferation and transformation [47]. Our observation that CCG-1423 suppresses E2F-modulated genes fits well with the recently defined ability of RhoA or C to induce E2F-regulated genes in NIH-3T3 cells [18]. Furthermore, the E2F-related gene clusters are also at least partially suppressed by Lat B which blocks Rho-induced gene expression by a different mechanism. Finally, the role of the E2F pathway in Rho-mediated transformation in the 3T3 system suggests that this will have important functional implications [18].

As noted in the Results Section, substantial information suggests relevance of our compound’s effects to melanoma. Microarray data sets from primary melanomas identified genes related to the propensity of a tumor to metastasize and/or relapse [6,48]. CCG-1423 reduced expression (in PC-3 cells) of those same gene sets. Whether those regulated genes are causal in the metastasis/relapse or are just markers, it will be of interest to determine whether compounds of this sort could have a beneficial effect in preclinical models. Even if those genes are markers, they are likely to be driven by a signaling mechanism or gene program that, if abrogated, could have a beneficial effect. Two key publications further support the importance of Rho/MKL/SRF signaling in melanoma metastasis. First, Hynes and colleagues found markedly increased RhoC expression in the A375M2 cell line, which had been selected by twice isolating pulmonary metastases of the parental A375 melanoma line. We previously demonstrated that A375M2 cells were especially sensitive to the growth inhibition and apoptosis-inducing effects of CCG-1423 [23]. More recently, Treisman and colleagues showed that MKL and SRF were critical for metastasis of BF16F2 melanoma cells [22]. This latter result is critical to the concept that Rho-stimulated gene transcription is a major element in cancer aggressiveness and metastasis. That was precisely the rationale for pursuing compounds with Rho transcription inhibition activity such as those in the CCG-1423 series.

Similar types of information implicate a role for Rho-regulated gene transcription in breast cancer. RhoC is highly over-expressed in inflammatory breast cancer, arguably the most aggressive form of breast cancer [49]. Also, RhoC^−/−^ mice show a much lower frequency of lung metastases from breast tumors caused by polyoma middle T-antigen expression despite similarly sized primary tumors [50]. Most relevant to Rho-stimulated gene transcription is the suppressing effect of MKL knock-down on metastasis of MDA-MB-231 xenografts [22]. We see a correlation of gene expression effects by CCG-1423 in the prostate cancer cells with the MKL-dependent genes from Treisman’s study of melanoma and breast cancer (Figure 2). Specifically MYH9 and MYL9 were among the few shared MKL-dependent genes in those two tumor types and our compound reduces expression of both (*ca.* 2–3-fold) in PC-3 cells. This suggests that CCG-1423 represses processes that are active in numerous cancer cell types.

There remain a number of questions to be answered regarding CCG-1423. What is the direct molecular target(s) of CCG-1423 and more recently identified analogs [45]. What is the significance of the “additional” genes modulated by the compound compared to the relatively smaller number of genes that appear to be MKL-dependent in the studies from Prywes [19] and Treisman [22]. E2F-regulated genes may play a key role in the action of CCG-1423 given their strong induction by RhoA and RhoC in NIH-3T3 cells [18]. It is not known, however, if those are actually MKL-dependent.

The ability of CCG-1423 to suppress expression of genes activated by the RhoA/C pathway confirms our previous conclusion that it is a Rho/MKL/SRF pathway inhibitor. Furthermore, the strong suppression of E2F-regulated genes provides additional rationale for its testing in aggressive cancer models. Future work remains to definitively identify the direct molecular target of this compound and to understand the mechanism behind the fairly broad scope of genes whose expression it modulates. Additional studies in melanoma and breast cancer, which are strongly influenced by RhoA/C pathways, as well as identification of the properties of tumor subsets that might make them particularly responsive to this family of compounds will be important future directions.

## 5. Conclusions

In this study, we have uncovered several key cellular gene expression networks that are perturbed by our compound CCG-1423, including cell cycle mediators, Rho-mediated gene expression, and ER stress mechanisms. In addition, modulation of MKL/SRF dependent metastasis networks in both breast cancer and melanoma cell lines with our compound highlights the importance of pharmacologic inhibitors of this pathway as potential future therapeutics in cancer biology.

## Figures and Tables

**Table 1:** Number of genes regulated by compounds.

Treatment	2 h			24 h		
	Stimulated	Inhibited	Total	Stimulated	Inhibited	Total
CCG-1423	48	1	49	837	1307	2142
Lat B	15	3	18	225	383	608
DRB	5	714	719	1058	2026	3020 *

This table shows the number of genes whose expression changed 2-fold after treatment of PC-3 prostate cancer cells under serum-starved conditions (0.5% FBS) with 3 µM CCG-1423, 0.5 µM Lat B, and 50 µM DRB. * The total number of genes changes does not equal the sum of stimulated and inhibited gene totals because some genes have probesets that are stimulated and others that are inhibited.

**Table 2:** Comparison of genes regulated by CCG-1423 with MKL1-dependent genes from the literature.

Gene Categories	CCG-1423	Lat B	DRB	References
Total Genes Regulated	2142	608	3020	
FBS-MKL1-Dependent Genes (#) (NIH3T3)	5 out of 28	0 out of 28	7 out of 28	[20]
FBS-MKL1-Dependent Genes (%) (NIH3T3)	17.8%	0.0%	25.0%	[20]
MKL1-Dependent Genes (#) (MDA-MB-231)	273 out of 1070	83 out of 1070	271 out of 1070	[22]
MKL1-Dependent Genes (%) (MDA-MB-231)	25.5%	7.8%	25.3%	[22]
MKL1-Dependent Genes (#) (B16F2)	73 out of 323	54 out of 323	58 out of 323	[22]
MKL1-Dependent Genes (%) (B16F2)	22.6%	16.7%	18.0%	[22]

Shown are the number and percentage of genes regulated by CCG-1423, Lat B, and DRB that share identity with genes regulated by serum (10% FBS)-induced in an MKL1-dependent manner in NIH3T3 mouse fibroblast cells [20] or those suppressed by knock-down of MTRF-A/B (*i.e.*, MKL1/2-dependent) in aggressive MDA-MB-231 breast cancer cells or B16F2 melanoma cells [22].

**Table 3:** Comparison of microarray *versus* qRT-PCR results.

Gene	Microarray	qRT-PCR
	(Fold-change)	(Fold-change)
*RGS4*	21.7, 21.6, 19.4 *	12.4
*RGS7*	9.6	9.9
*CTGF*	0.05	0.12
*SOX9*	0.12, 0.13 *	0.19

PC-3 cells were treated with 3 µM of CCG-1423 under serum-starved conditions (0.5% FBS) for 24 h and gene expression was assessed with the Affymetrix array or in a quantitative real-time-PCR (qRT-PCR) reaction assay. The genes RGS4, RGS7, CTGF, and SOX9, which were identified as candidates, were confirmed using a qRT-PCR assay. The mean fold-change values (over DMSO Control) for expression of these genes (*n* = 3) are displayed in the table. For the Microarray data, the log2 transformed expression values were retransformed by exponentiating using 2*x*, where *x* equals the log2 expression value. Then, the fold-change values were calculated by dividing the expression values for the CCG-1423-treated samples by the expression values for the DMSO samples. * indicates mean fold-change values of different probesets for RGS4 or SOX9.

## Supplementary Materials

The following are available online at http://www.mdpi.com/2076-3905/5/2/13/s1.

## Abbreviations

The following abbreviations are used in this manuscript:

MKL	Megakaryocytic Leukemia
SRF	Serum Response Factor
Lat B	Latrunculin B
DRB	5,6-dichloro-1-β-d-ribofuranosyl-1H-benzimidazole
ER	Endoplasmic reticulum
