# Supplementary material for: Small-Molecule Inhibition of Rho/MKL/SRF Transcription in Prostate Cancer Cells: Modulation of Cell Cycle, ER Stress, and Metastasis Gene Networks
Source: Microarrays (Basel). 2016 May 28;5(2):13. doi: 10.3390/microarrays5020013 (PMC5003489; doi:10.3390/microarrays5020013)
Supplement: Supplementary file 1 [file microarrays-05-00013-s001.pdf]

Supplementary Materials: Small-Molecule Inhibition of Rho/MKL/SRF Transcription in Prostate Cancer Cells: Modulation of Cell Cycle, ER Stress, and Metastasis Gene Networks

Chris R. Evelyn, Erika M. Lisabeth, Susan M. Wade, Andrew J. Haak, Craig N. Johnson, Elizabeth R. Lawlor and Richard R. Neubig

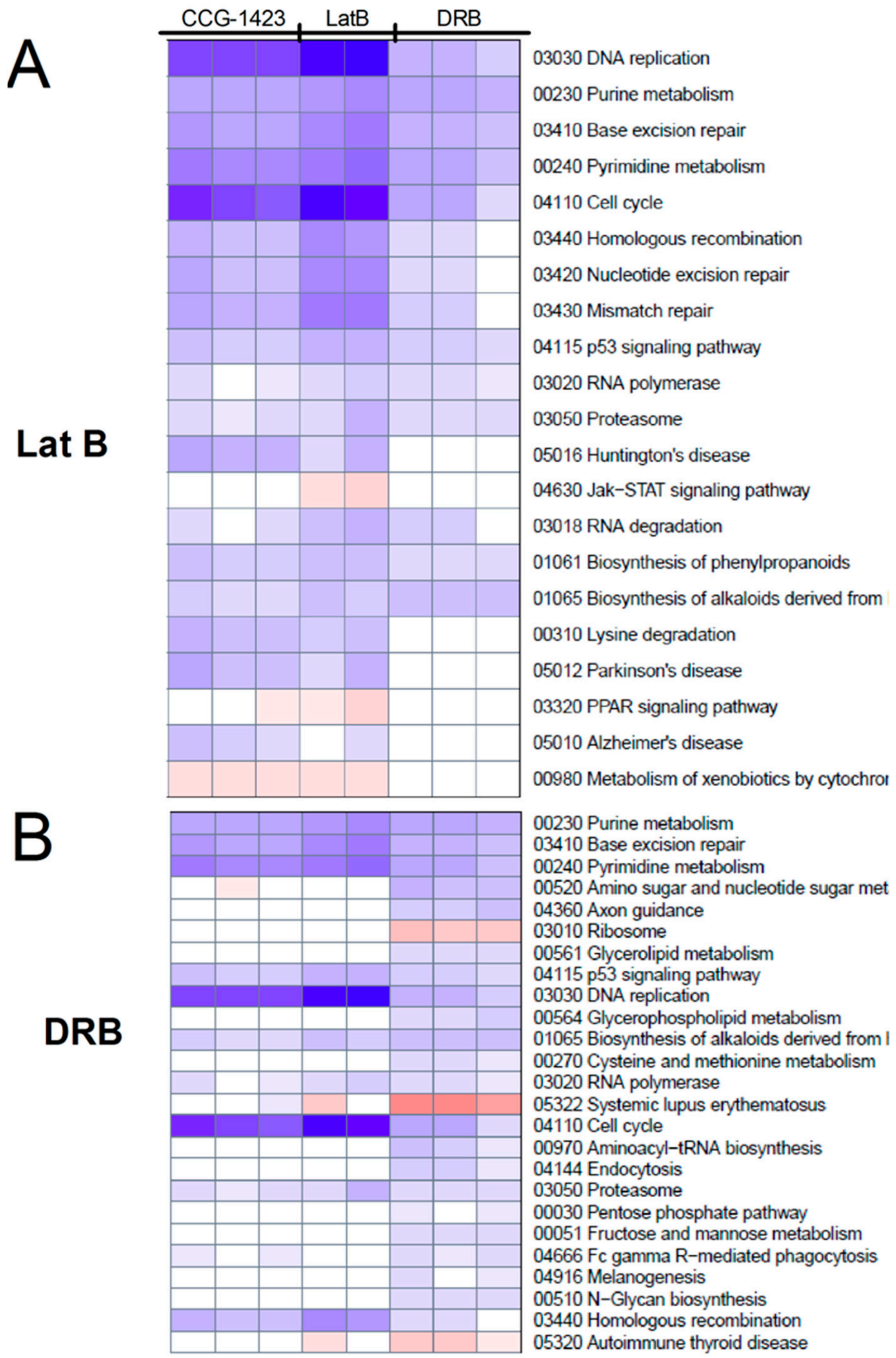

Figure S1. GSEA KEGG categories for gene families modulated by Lat B and DRB. The gene sets for different KEGG categories that were significantly associated with those altered by Lat B and DRB are illustrated as shown for CCG-1423 in Figure 4.

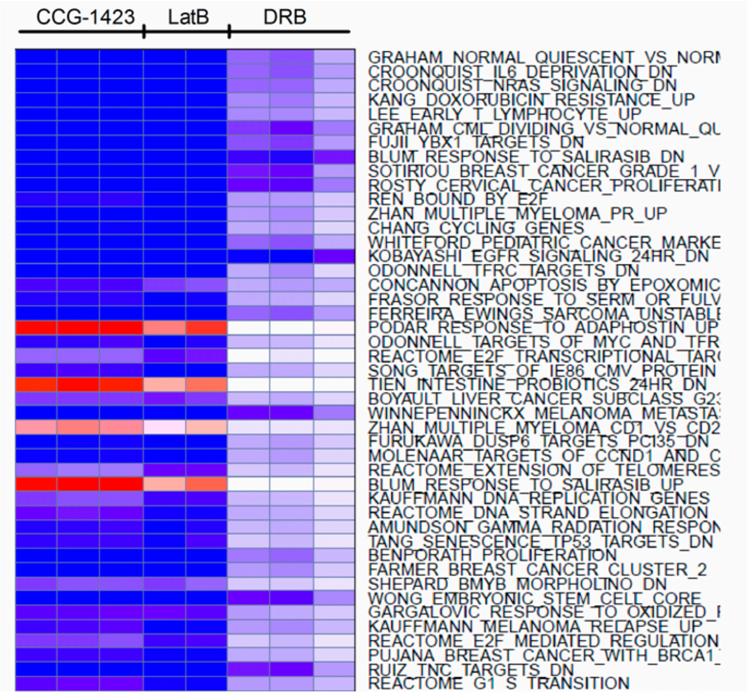

Figure S2. GSEA analysis for comparison to MSigDB experimental gene sets. Experimental gene sets of the MSigDB (Broad Version 3.0) were examined using GSEA and the entire microarray data set. The top 25 gene sets significantly associated with changes induced by CCG-1423 (3 μM) are shown along with the related changes induced by Lat B (0.5 μM) and DRB (30 μM). See text and Table S4 for additional information. Colors represent *Z*-scores with Blue indicating genes of the set were downregulated and red indicating genes of the set were up-regulated. The darkness represents the level of significance. White means that the adjusted *p* value is >0.05.

Table S1. CCG-1423 and Lat B regulated genes (2 h). Blue highlighted gene transcripts indicate genes stimulated by CCG-1423, and red highlighted gene transcripts indicate genes inhibited by CCG-1423.

Symbol	Description	GenBank	Fold Change (Log2 Values)
	**CCG-1423 (2 h)**		
ATF3	Activating transcription factor 3	NM_001674	−2.95
IL8	Interleukin 8	AF043337	−2.47
		BF111326	−2.33
KCNJ2	Potassium inwardly-rectifying channel, subfamily J, member 2	AF153820	−2.17
ERRFI1	ERBB receptor feedback inhibitor 1	AW612461	−2.11
CHAC1	ChaC, cation transport regulator homolog 1 (*E. coli*)	NM_024111	−2.09
PDK4	Pyruvate dehydrogenase kinase, isozyme 4	AV707102	−1.96
AREG	Amphiregulin (schwannoma-derived growth factor)	NM_001657	−1.88
GDF15	Growth differentiation factor 15	AF003934	−1.77
MYC	v-Myc myelocytomatosis viral oncogene homolog (avian)	NM_002467	−1.77
TMEM49	Transmembrane protein 49	AL541655	−1.61
PPP1R15A	Protein phosphatase 1, regulatory (inhibitor) subunit 15A	NM_014330	−1.38
RASD1	RAS, dexamethasone-induced 1	AF069506	−1.37
DDIT3	DNA-damage-inducible transcript 3	BC003637	−1.37
IL1B	Interleukin 1, beta	M15330	−1.37
FBXO32	F-box protein 32	AW006123	−1.35
CXCL1	Chemokine (C-X-C motif) ligand 1 (melanoma growth stimulating activity, alpha)	NM_001511	−1.33
AMOTL2	Angiomotin like 2	NM_016201	−1.32
ZEB1	Zinc finger E-box binding homeobox 1	NM_030751	−1.29
SEMA3C	Sema domain, immunoglobulin domain (Ig), short basic domain, secreted, (semaphorin) 3C	R62588	−1.22
KLF4	Kruppel-like factor 4 (gut)	BF514079	−1.21
TIPARP	TCDD-inducible poly(ADP-ribose) polymerase	AL556438	−1.2
NEDD9	Neural precursor cell expressed, developmentally down-regulated 9	U64317	−1.19
SLC3A2	Solute carrier family 3 (activators of dibasic and neutral amino acid transport), member 2	NM_002394	−1.18
STK17B	Serine/threonine kinase 17b	N51102	−1.16
LOC727738	Similar to Amphiregulin precursor (AR) (Colorectum cell-derived growth factor) (CRDGF)	AI891075	−1.16
RND3	Rho family GTPase 3	BG054844	−1.09

Table S1. *Cont.*

Symbol	Description	GenBank	Fold Change (Log_2 Values)
	**CCG-1423 (2 h)**		
GADD45A	Growth arrest and DNA-damage-inducible, alpha	NM_001924	−1.09
GEM	GTP binding protein overexpressed in skeletal muscle	NM_005261	−1.09
SQSTM1	Sequestosome 1	AW293441	−1.09
SLC19A2	Solute carrier family 19 (thiamine transporter), member 2	AF153330	−1.09
JUN	Jun oncogene	NM_002228	−1.08
PIM1	Pim-1 oncogene	M24779	−1.07
DDIT4	DNA-damage-inducible transcript 4	NM_019058	−1.07
CTGF	Connective tissue growth factor	M92934	−1.06
HBEGF	Heparin-binding EGF-like growth factor	M60278	−1.05
ADFP	Adipose differentiation-related protein	BC005127	−1.05
CYR61	Cysteine-rich, angiogenic inducer, 61	NM_001554	−1.05
INHBA	Inhibin, βA	M13436	−1.05
RP5-1022P6.2	Hypothetical protein KIAA1434	AK001947	−1.04
ADRB2	Adrenergic, β-2-, receptor, surface	NM_000024	−1.02
DCUN1D3	DCN1, defective in cullin neddylation 1, domain containing 3 (*S. Cerevisiae*)	AI765327	−1.01
SAMD4A	Sterile alpha motif domain containing 4A	AW802645	−1.01
TUFT1	Tuftelin 1	NM_020127	−1.01
KLF5	Kruppel-like factor 5 (intestinal)	AF132818	−1.01
SOX7	SRY (sex determining region Y)-box 7	BC004299	−1.01
THBS1	Thrombospondin 1	AV726673	−1
CXCL2	Chemokine (C-X-C motif) ligand 2	M57731	−1
LOC284801	Hypothetical protein LOC284801	AL531683	1.18
	**Lat B (2 h)**		
		N21643	−1.62
ITPKB	Inositol 1,4,5-trisphosphate 3-kinase B	AA348410	−1.56
KLF6	Kruppel-like factor 6	BE675435	−1.49
FBXO32	F-box protein 32	AW006123	−1.45
ADAMTS1	ADAM metallopeptidase with thrombospondin type 1 motif, 1	AK023795	−1.34

Table S1. *Cont.*

Symbol	Description	GenBank	Fold Change (Log2 Values)
	**Lat B (2 h)**		
DUSP10	Dual specificity phosphatase 10	N36770	−1.32
ERRFI1	ERBB receptor feedback inhibitor 1	AW612461	−1.32
NDRG1	N-myc downstream regulated gene 1	AI732596	−1.28
GEM	GTP binding protein overexpressed in skeletal muscle	NM_005261	−1.26
CD55	CD55 molecule, decay accelerating factor for complement (Cromer blood group)	AI679555	−1.24
ID2	Inhibitor of DNA binding 2, dominant negative helix-loop-helix protein	AI819238	−1.16
IRS2	Insulin receptor substrate 2	BF700086	−1.14
JUN	Jun oncogene	BG491844	−1.06
GDF15	Growth differentiation factor 15	AF003934	−1.03
FLRT3	Fibronectin leucine rich transmembrane protein 3	N71923	−1
FLNB	Filamin B, β (actin binding protein 278)	AI524619	1
FOS	v-Fos FBJ murine osteosarcoma viral oncogene homolog	BC004490	1.02
CYR61	Cysteine-rich, angiogenic inducer, 61	NM_001554	1.24

Table S2. Metastasis-Related "Candidate" Gene Transcripts. The 2142 genes changed by CCG-1423 were filtered using metastasis-related gene ontology (GO) categories, including: angiogenesis, cell migration, cell adhesion, epithelial-mesenchymal-transition, extracellular matrix, G-protein coupled receptor protein signaling pathway, inflammatory response, and metalloendopeptidases. This table lists the 203 unique metastasis-related genes identified using this criteron. For several genes, there are multiple probesets. Blue highlighted gene transcripts indicate genes stimulated by CCG-1423, and red highlighted gene transcripts indicate genes inhibited by CCG-1423.

Symbol	Description	GenBank	Fold Change (Log2 Values)
S100P	S100 Calcium Binding Protein P	NM_005980	−5.40
RGS4	Regulator of G-Protein Signaling 4	BC000737	−4.28
RGS4	Regulator of G-Protein Signaling 4	AL514445	−4.44
RGS4	Regulator of G-Protein Signaling 4	NM_005613	−4.43
CXCL2	Chemokine (C-X-C motif) Ligand 2	M57731	−4.12
IL8	Interleukin 8	AF043337	−3.89

Table S2. *Cont.*

Symbol	Description	GenBank	Fold Change (Log2 Values)
IL8	Interleukin 8	NM_000584	−3.81
PTGS2	Prostaglandin-Endoperoxide Synthase 2 (Prostagalndin G/H Synthase and Cyclooxygenase)	AY151286	−3.83
PTGS2	Prostaglandin-Endoperoxide Synthase 2 (Prostagalndin G/H Synthase and Cyclooxygenase	NM_000963	−5.23
HMOX1	Heme Oxygenase (Decycling) 1	NM_002133	−3.59
ADAMTS5	ADAM Metallopeptidase withThrombospondin Type 1 Motif, 5 (Aggrecanase-2)	NM_007038	−3.31
ADAMTS5	ADAM Metallopeptidase withThrombospondin Type 1 Motif, 5 (Aggrecanase-2)	AI123555	−2.88
ADAMTS5	ADAM Metallopeptidase withThrombospondin Type 1 Motif, 5 (Aggrecanase-2)	BF060767	−2.74
ADAMTS5	ADAM Metallopeptidase withThrombospondin Type 1 Motif, 5 (Aggrecanase-2)	BI254089	−1.11
RGS7	Regulator of G-Protein Signaling 7	NM_002924	−3.27
HGF	Hepatocyte Growth Factor (Hepapoietin A; Scatter Factor)	X16323	−3.26
HGF	Hepatocyte Growth Factor (Hepapoietin A; Scatter Factor)	M77227	−2.91
HGF	Hepatocyte Growth Factor (Hepapoietin A; Scatter Factor)	U46010	−1.72
HGF	Hepatocyte Growth Factor (Hepapoietin A; Scatter Factor)	M60718	−1.57
HGF	Hepatocyte Growth Factor (Hepapoietin A; Scatter Factor)	M77227	−1.81
MFAP2	Microfibrillar-Associated Protein 2	NM_017459	−2.76
L1CAM	L1 Cell Adhesion Molecule	AI653981	−2.75
DST	Dystonin	NM_001723	−2.70
CXCL3	Chemokine (C-X-C Motif) Ligand 3	NM_002090	−2.69
MMP10	Matrix Metallopeptidase 10 (Stromelysin 2)	NM_002425	−2.68
CEBPB	CCAAT/Enhancer Binding Protein (C/EBP), Beta	AL564683	−2.66
ANXA1	Annexin A1	AU155094	−2.60

Table S2. *Cont.*

Symbol	Description	GenBank	Fold Change (Log2 Values)
ANXA1	Annexin A1	NM_000700	−1.87
VEGFA	Vascular Endothelial Growth Factor A	M27281	−2.60
VEGFA	Vascular Endothelial Growth Factor A	H95344	−2.49
VEGFA	Vascular Endothelial Growth Factor A	AF022375	−2.43
VEGFA	Vascular Endothelial Growth Factor A	AF091352	−2.76
RND3	Rho Family GTPase 3	BG054844	−2.58
LAMA3	Laminin, Alpha 3	BC033663	−2.35
LAMA3	Laminin, Alpha 3	AK024889	−1.11
ADRB1	Adrenergic, Beta-1-, Receptor	AI625747	−2.34
CDH7	Cadherin 7, Type 2	NM_004361	−2.20
ADAM17	ADAM Metallopeptidase Domain 17 (Tumor Necrosis Factor, α, Converting Enzyme)	U86755	−2.11
ADAM17	ADAM Metallopeptidase Domain 17 (Tumor Necrosis Factor, α, Converting Enzyme)	NM_003183	−1.98
FGF2	Fibroblast Growth Factor 2 (Basic)	M27968	−2.10
FGF2	Fibroblast Growth Factor 2 (Basic)	NM_002006	−1.83
IL6	Interleukin 6 (Interferon, β2)	NM_000600	−2.02
ELF3	E74-Like Factor 3 (ETS Domain Transcription Factor, Epithelial-Specific)	U73844	−1.96
ELF3	E74-Like Factor 3 (ETS Domain Transcription Factor, Epithelial-Specific)	AA527180	−1.73
ELF3	E74-Like Factor 3 (ETS Domain Transcription Factor, Epithelial-Specific)	AF017307	−1.86
HPSE	Heparanase	AF155510	−1.94
HPSE	Heparanase	NM_006665	−1.03
TNFAIP6	Tumor Necrosis Factor, Alpha-Induced Protein 6	NM_007115	−1.91
TNFAIP6	Tumor Necrosis Factor, Alpha-Induced Protein 6	AW188198	−2.05
CLDN12	Claudin 12	AL136770	−1.77

Table S2. *Cont.*

Symbol	Description	GenBank	Fold Change (Log2 Values)
RTN4	Reticulon 4	N90377	−1.74
RTN4	Reticulon 4	CA428769	−1.42
RTN4	Reticulon 4	AW963634	−1.14
FLRT3	Fibronectin Leucine Rich Transmembrane Protein 3	N71923	−1.69
FLRT3	Fibronectin Leucine Rich Transmembrane Protein 3	NM_013281	−1.83
ITGAV	Integrin, Alpha V (Vitronectin Receptor, Alpha Polypeptide, Antigen CD51)	AA228366	−1.67
ITGAV	Integrin, Alpha V (Vitronectin Receptor, Alpha Polypeptide, Antigen CD51)	AW962458	−1.43
ITGAV	Integrin, Alpha V (Vitronectin Receptor, Alpha Polypeptide, Antigen CD51)	AU144005	−1.13
LY96	Lymphocyte Antigen 96	NM_015364	−1.63
CDH1	Cadherin 1, Type 1, E-Cadherin (Epithelial)	L08599	−1.61
CDH1	Cadherin 1, Type 1, E-Cadherin (Epithelial)	NM_004360	−1.61
IRAK2	Interleukin-1 Receptor-Associated Kinase 2	AI246590	−1.59
LUM	Lumican	NM_002345	−1.57
OPN3	Opsin 3 (Encephalopsin, Panopsin)	AI074145	−1.56
C3	Complement Component 3	NM_000064	−1.52
NFE2L1	Nuclear Factor (Erythroid-Derived 2)-Like 1	H93013	−1.52
NFE2L1	Nuclear Factor (Erythroid-Derived 2)-Like 1	NM_003204	−1.44
NFE2L1	Nuclear Factor (Erythroid-Derived 2)-Like 1	AI361227	−1.13
ZFP36	Zinc Finger Protein 36, C3H Type, Homolog (Mouse)	NM_003407	−1.52
CLDN14	Claudin 14	AF314090	−1.51
CCRL2	Chemokine (C-C Motif) Receptor-Like 2	AF015524	−1.50
AKAP12	A Kinase (PRKA) Anchor Protein (Gravin) 12	BF511276	−1.48
AKAP12	A Kinase (PRKA) Anchor Protein (Gravin) 12	AI672553	−1.46
AKAP12	A Kinase (PRKA) Anchor Protein (Gravin) 12	BF511276	−1.35
AKAP12	A Kinase (PRKA) Anchor Protein (Gravin) 12	AB003476	−1.13
KLF5	Kruppel-Like Factor 5 (Intestinal)	AB030824	−1.45
KLF5	Kruppel-Like Factor 5 (Intestinal)	AF132818	−1.64

Table S2. *Cont.*

Symbol	Description	GenBank	Fold Change (Log_2 Values)
BTG1	B-Cell Translocation Gene 1, Anti-Proliferative	AL535380	−1.44
BTG1	B-Cell Translocation Gene 1, Anti-Proliferative	NM_001731	−1.49
TPBG	Trophoblast Glycoprotein	NM_006670	−1.43
ACVR1	Activin A Receptor, Type I	NM_001105	−1.41
EDNRA	Endothelin Receptor Type A	NM_001957	−1.39
COL5A1	Collagen, Type V, Alpha 1	N30339	−1.36
COL5A1	Collagen, Type V, Alpha 1	AI983428	−1.06
WNT9A	Wingless-Type MMTV Integration Site Family, Member 9A"	BE220265	−1.34
ALCAM	Activated Leukocyte Cell Adhesion Molecule	BE502785	−1.31
ALCAM	Activated Leukocyte Cell Adhesion Molecule	BC041127	−1.33
NOS3	Nitric Oxide Synthase 3 (Endothelial Cell)	NM_000603	−1.31
CLDN7	Claudin 7	NM_001307	−1.29
BMP6	Bone Morphogenetic Protein 6	NM_001718	−1.26
GPC1	Glypican 1	NM_002081	−1.25
ERAP1	Endoplasmic Reticulum Aminopeptidase 1	BE551138	−1.24
PCDH7	Protocadherin 7	BE644809	−1.24
ADAMTS17	ADAM Metallopeptidase with Thrombospondin Type 1 Motif, 17	AA022668	−1.23
ADAMTS17	ADAM Metallopeptidase with Thrombospondin Type 1 Motif, 17	AA022668	−1.01
RRAS2	Related RAS Viral (R-Ras) Oncogene Homolog 2	NM_012250	−1.21
RRAS2	Related RAS Viral (R-Ras) Oncogene Homolog 2	AI753792	−1.18
RRAS2	Related RAS Viral (R-Ras) Oncogene Homolog 2	AI431643	−1.10
CD24	CD24 Molecule	AA761181	−1.17
CD24	CD24 Molecule	L33930	−1.32
CD24	CD24 Molecule	AK000168	−1.18
CD24	CD24 Molecule	X69397	−1.09
CD24	CD24 Molecule	M58664	−1.18
CD24	CD24 Molecule	BG327863	−1.06

Table S2. *Cont.*

Symbol	Description	GenBank	Fold Change (Log_2 Values)
CELSR1	Cadherin, EGF LAG Seven-Pass G-Type Receptor 1 (Flamingo Homolog, Drosophila)	AL031588	−1.17
MPZL3	Myelin Protein Zero-Like 3	AA772172	−1.16
CEACAM1	Carcinoembryonic Antigen-Related Cell Adhesion Molecule 1 (BiliaryGlycoprotein)	M76742	−1.15
CEACAM1	Carcinoembryonic Antigen-Related Cell Adhesion Molecule 1 (BiliaryGlycoprotein)	X16354	−1.09
AGT	Angiotensinogen (Serpin Peptidase Inhibitor, Clade A, Member 8)	NM_000029	−1.14
TYMP	Thymidine Phosphorylase	NM_001953	−1.14
TYMP	Thymidine Phosphorylase	AW613387	−1.25
AMIGO2	Adhesion Molecule with Ig-Like Domain 2	AC004010	−1.12
RHOB	RAS Homolog Gene Family, Member B	AI263909	−1.12
HDAC9	Histone Deacetylase 9	NM_014707	−1.10
GPR92	G-Protein Coupled Receptor 92	AW183080	−1.09
OR2A7	Olfactory Receptor, Family 2, Subfamily A, Member 7	AF327904	−1.09
BMP2	Bone Morphogenetic Protein 2	NM_001200	−1.04
CD58	CD58 Molecule	D28586	−1.04
ITGA2	Integrin, Alpha 2 (CD49B, Alpha 2 Subunit of VLA-2 Receptor)	N95414	−1.03
LAMA4	Laminin, Alpha 4	U77706	−1.03
SERPINE2	Serpin Peptidase Inhibitor, Clade E (Nexin, Plasminogen Activator Inhibitor Type 1), Member 2	AL541302	−1.03
SERPINE2	Serpin Peptidase Inhibitor, Clade E (Nexin, Plasminogen Activator Inhibitor Type 1), Member 2	AA703280	−1.49
DSG2	Desmoglein 2	BF031829	−1.02
FERMT2	Fermitin Family Homolog 2 (Drosophila)	Z24725	−1.01
FERMT2	Fermitin Family Homolog 2 (Drosophila)	AW469573	−1.04
SCYL3	SCY1-Like 3 (S. Cerevisiae)	AI458463	−1.01
PDGFRA	Platelet-Derived Growth Factor Receptor, Alpha Polypeptide	NM_006206	1.00

Table S2. *Cont.*

Symbol	Description	GenBank	Fold Change (Log_2 Values)
EDG2	Endothelial Differentiation, Lysophosphatidic Acid G-Protein-Coupled Receptor, 2	AW269335	1.01
FN1	Fibronectin 1	AJ276395	1.01
SLIT2	Slit Homolog 2 (Drosophila)	AI692523	1.01
TGFB2	Transforming Growth Factor, Beta 2	M19154	1.01
TGFB3	Transforming Growth Factor, Beta 3	J03241	1.01
JUB	JUB, AJUBA Homolog (Xenopus Laevis)	NM_032876	1.02
TFPI2	Tissue Factor Pathway Inhibitor 2	L27624	1.02
CDK5	Cyclin-Dependent Kinase 5	NM_004935	1.03
MGLL	Monoglyceride Lipase	BC006230	1.03
ABI2	ABL Interactor 2	BF245400	1.04
NTNG1	Netrin G1	AV723308	1.04
SSX2IP	Synovial Sarcoma, X Breakpoint 2 Interacting Protein	R52678	1.04
TUBB3	Tubulin, Beta 3	NM_006086	1.04
TUBB3	Tubulin, Beta 3	AL565749	1.05
FZD4	Frizzled Homolog 4 (Drosophila)	NM_012193	1.05
FZD4	Frizzled Homolog 4 (Drosophila)	AB054881	1.15
GNAS	GNAS Complex Locus	AA401492	1.05
LOXL2	Lysyl Oxidase-Like 2	NM_002318	1.07
BAI2	Brain-Specific Angiogenesis Inhibitor 2	NM_001703	1.08
ITGB1	Integrin, Beta 1 (Fibronectin Receptor, Beta Polypeptide, Antigen CD29 includes MDF2, MSK12)	AF086249	1.08
NARG1	NMDA Receptor Regulated 1	NM_025085	1.08
LAMB1	Laminin, Beta 1	M20206	1.09
LAMB1	Laminin, Beta 1	NM_002291	1.12
ATPIF1	ATPase Inhibitory Factor 1	NM_016311	1.11
ROBO1	Roundabout, Axon Guidance Receptor, Homolog 1 (Drosophila)	BF059159	1.11
CCDC88A	Coiled-Coil Domain Containing 88A	AB033038	1.12
ABHD2	Abhydrolase Domain Containing 2	BE671816	1.13

Table S2. *Cont.*

Symbol	Description	GenBank	Fold Change (Log_2 Values)
ABHD2	Abhydrolase Domain Containing 2	AI832249	1.12
CHST2	Carbohydrate (N-acetylglucosamine-6-O) Sulfotransferase 2	NM_004267	1.14
ITGB2	Integrin, Beta 2 (Complement Component 3 Receptor 3 and 4 Subunit)	NM_000211	1.15
ITGB2	Integrin, Beta 2 (Complement Component 3 Receptor 3 and 4 Subunit)	AW303397	1.38
PTGER4	Prostaglandin E Receptor 4 (Subtype EP4)	AA897516	1.15
ITGAE	Integrin, Alpha E (Antigen CD103, Human Mucosal Lymphocyte Antigen 1; Alpha Polypeptide)	NM_002208	1.17
VEGFC	Vascular Endothelial Growth Factor C	U58111	1.17
GAL	Galanin Prepropeptide	AL556409	1.19
GPSM2	G-Protein Signaling Modulator 2 (AGS3-like, C. Elegans)	NM_013296	1.19
GPSM2	G-Protein Signaling Modulator 2 (AGS3-like, C. Elegans)	AW195581	1.15
TRIP6	Thyroid Hormone Receptor Interactor 6	AF000974	1.19
ITGA10	Integrin, Alpha 10	AF112345	1.20
CD47	CD47 Molecule	AL118798	1.21
COL27A1	Collagen, Type XXVII, Alpha 1	AK021957	1.23
MAGI1	Membrane Associated Guanylate Kinase, WW and PDZ Domain Containing 1	AI141556	1.23
COCH	Coagulation Factor C Homolog, cochlin (Limulus Polyphemus)	AA669336	1.24
GNG2	Guanine Nucleotide Binding Protein (G protein), Gamma 2	AU118419	1.24
GNG2	Guanine Nucleotide Binding Protein (G protein), Gamma 2	AK026424	1.18
GNG2	Guanine Nucleotide Binding Protein (G protein), Gamma 2	AF493870	1.05
ADAM9	ADAM Metallopeptidase Domain 9 (Meltrin Gamma)	AF495383	1.25
ADAM9	ADAM Metallopeptidase Domain 9 (Meltrin Gamma)	NM_003816	1.31
CDH3	Cadherin 3, Type 1, P-Cadherin (Placental)	NM_001793	1.29
CYFIP2	Cytoplasmic FMR1 Interacting Protein 2	AL161999	1.31
PXN	Paxillin	NM_002859	1.31
PXN	Paxillin	D86862	1.74
GPER	G Protein-Coupled Estrogen Receptor 1	U58828	1.32

Table S2. *Cont.*

Symbol	Description	GenBank	Fold Change (Log_2 Values)
GPER	G Protein-Coupled Estrogen Receptor 1	U63917	1.29
PDZD2	PDZ Domain Containing 2	AF338650	1.33
BMPR1B	Bone Morphogenetic Protein Receptor, Type IB	D89675	1.34
ITGA6	Integrin, Alpha 6	AV733308	1.35
MYH9	Myosin, Heavy Chain 9, Non-Muscle	AI827941	1.35
COL12A1	Collagen, Type XII, Alpha 1	AL096771	1.36
COL12A1	Collagen, Type XII, Alpha 1	AA788946	1.49
COL12A1	Collagen, Type XII, Alpha 1	U73778	1.08
EPHX2	Epoxide Hydrolase 2, Cytoplasmic	AF233336	1.37
TGM2	Transglutaminase 2 (C Polypeptide, Protein-Glutamine-Gamma-Glutamyltransferase)	BC003551	1.38
TGM2	Transglutaminase 2 (C Polypeptide, Protein-Glutamine-Gamma-Glutamyltransferase)	AL031651	1.65
TGM2	Transglutaminase 2 (C Polypeptide, Protein-Glutamine-Gamma-Glutamyltransferase)	M98478	1.08
GRK5	G Protein-Coupled Receptor Kinase 5	NM_005308	1.42
ARHGDIB	Rho GDP Dissociation Inhibitor (GDI) Beta	NM_001175	1.44
CDH11	Cadherin 11, Type 2, OB-Cadherin (Osteoblast)	NM_001797	1.45
CDH11	Cadherin 11, Type 2, OB-Cadherin (Osteoblast)	D21254	1.34
CDH11	Cadherin 11, Type 2, OB-Cadherin (Osteoblast)	AI040305	1.08
EDG3	Endothelial Differentiation, Sphingolipid G-Protein-Coupled Receptor, 3	AA534817	1.46
C21orf29	Chromosome 21 Open Reading Frame 29	BC021197	1.47
GBX2	Gastrulation Brain Homeobox 2	AF118452	1.48
PI3	Peptidase Inhibitor 3, Skin-Derived (SKALP)	L10343	1.49
PI3	Peptidase Inhibitor 3, Skin-Derived (SKALP)	NM_002638	1.44
ITGA1	Integrin, Alpha 1	X68742	1.51
SSPN	Sarcospan (Kras Oncogene-Associated Gene)	AW467136	1.52
SSPN	Sarcospan (Kras Oncogene-Associated Gene)	AL136756	1.30
SSPN	Sarcospan (Kras Oncogene-Associated Gene)	NM_005086	1.27
CALM3	Calmodulin 3 (Phosphorylase Kinase, Delta)	NM_005184	1.54

Table S2. *Cont.*

Symbol	Description	GenBank	Fold Change (Log2 Values)
CALM3	Calmodulin 3 (Phosphorylase Kinase, Delta)	AV685208	1.16
GNG11	Guanine Nucleotide Binding Protein (G protein), gamma 11	NM_004126	1.55
ACHE	Acetylcholinesterase (Yt Blood Group)	NM_015831	1.56
FBN2	Fibrillin 2 (Congenital Contractural Arachnodactyly)	NM_001999	1.59
JAG1	Jagged 1 (Alagille Syndrome)	U73936	1.59
JAG1	Jagged 1 (Alagille Syndrome)	U77914	1.79
JAG1	Jagged 1 (Alagille Syndrome)	AI457817	1.37
JAG1	Jagged 1 (Alagille Syndrome)	U61276	2.01
JAG1	Jagged 1 (Alagille Syndrome)	AU151465	1.19
COL4A1	Collagen, Type IV, Alpha 1	AI922605	1.61
COL4A1	Collagen, Type IV, Alpha 1	NM_001845	1.28
SERPINE1	Serpin Peptidase Inhibitor, Clade E (Nexin, Plasminogen Activator Inhibitor Type 1), Member 1	NM_000602	1.61
SERPINE1	Serpin Peptidase Inhibitor, Clade E (Nexin, Plasminogen Activator Inhibitor Type 1), Member 1	AL574210	1.39
FBLN1	Fibulin 1	NM_006486	1.62
FBLN1	Fibulin 1	Z95331	1.18
DSC3	Desmocollin 3	NM_001941	1.67
DSC3	Desmocollin 3	AI797281	1.43
MMP13	Matrix Metallopeptidase 13 (Collagenase 3)	NM_002427	1.67
NEGR1	Neuronal Growth Regulator 1	AA115106	1.67
NEGR1	Neuronal Growth Regulator 1	AI123532	2.52
NEGR1	Neuronal Growth Regulator 1	NM_173808	1.40
CNTNAP3	Contactin Associated Protein-Like 3	AF333769	1.68
CNTNAP3	Contactin Associated Protein-Like 3	AI433163	1.25
SPOCK1	Sparc/Osteonectin, CWCV and Kazal-Like Domains Proteoglycan (Testican) 1	AF231124	1.70
NEXN	Nexilin (F Actin Binding Protein)	AF114264	1.71

Table S2. *Cont.*

Symbol	Description	GenBank	Fold Change (Log_2 Values)
NEXN	Nexilin (F Actin Binding Protein)	NM_144573	1.92
PGM5	Phosphoglucomutase 5	AA706788	1.72
GPRC5B	G Protein-Coupled Receptor, Family C, Group 5, Member B	NM_016235	1.73
RGS2	Regulator of G-Protein Signaling 2, 24 kDa	NM_002923	1.77
GPR4	G Protein-Coupled Receptor 4	NM_005282	1.78
GPR4	G Protein-Coupled Receptor 4	U35399	1.15
COL6A3	Collagen, Type VI, Alpha 3	NM_004369	1.80
FABP4	Fatty Acid Binding Protein 4, Adipocyte	NM_001442	1.80
FAT	FATTumor Suppressor Homolog 1 (Drosophila)	NM_005245	1.86
LTBP1	Latent Transforming Growth Factor Beta Binding Protein 1	AI986120	1.86
LTBP1	Latent Transforming Growth Factor Beta Binding Protein 1	NM_000627	1.19
COL4A2	Collagen, Type IV, Alpha 2	X05610	1.90
COL4A2	Collagen, Type IV, Alpha 2	AA909035	2.12
TROAP	Trophinin Associated Protein (Tastin)	NM_005480	1.93
TROAP	Trophinin Associated Protein (Tastin)	AI199355	1.48
ITGB3BP	Integrin Beta 3 Binding Protein (Beta3-Endonexin)	NM_014288	1.95
CTNNAL1	Catenin (Cadherin-Associated Protein), Alpha-Like 1	NM_003798	2.01
PCDH20	Protocadherin 20	AA040057	2.02
COL5A2	Collagen, Type V, Alpha 2	NM_000393	2.03
COL5A2	Collagen, Type V, Alpha 2	AL575735	2.01
KAL1	Kallmann Syndrome 1 Sequence	NM_000216	2.03
AOX1	Aldehyde Oxidase 1	AB046692	2.05
AOX1	Aldehyde Oxidase 1	NM_001159	1.97
HAPLN1	Hyaluronan and Proteoglycan Link Protein 1	U43328	2.08
LOXL1	Lysyl Oxidase-Like 1	NM_005576	2.13
MCAM	Melanoma Cell Adhesion Molecule	BC006329	2.19
RELN	Reelin	BC041378	2.20

Table S2. *Cont.*

Symbol	Description	GenBank	Fold Change (Log2 Values)
RELN	Reelin	BC041378	2.00
ELK3	ELK3, ETS-Domain Protein (SRF Accessory Protein 2)	AW575374	2.23
ELK3	ELK3, ETS-Domain Protein (SRF Accessory Protein 2)	NM_005230	1.53
CYR61	Cysteine-Rich, Angiogenic Inducer, 61	NM_001554	2.24
CYR61	Cysteine-Rich, Angiogenic Inducer, 61	AF003114	2.38
P2RY5	Purinergic Receptor P2Y, G-Protein Coupled, 5	NM_005767	2.24
HSPG2	Heparan Sulfate Proteoglycan 2	M85289	2.26
HSPG2	Heparan Sulfate Proteoglycan 2	AI991033	1.90
NTSR1	Neurotensin Receptor 1 (High Affinity)	NM_002531	2.27
PCDH18	Protocadherin 18	AW189885	2.27
LRP5	Low Density Lipoprotein Receptor-Related Protein 5	AB017498	2.33
IGFBP4	Insulin-Like Growth Factor Binding Protein 4	NM_001552	2.35
IL23A	Interleukin 23, Alpha Subunit p19	AF043179	2.37
IL23A	Interleukin 23, Alpha Subunit p19	M15564	1.60
IL23A	Interleukin 23, Alpha Subunit p19	AL559122	1.13
CSGALNACT1	Chondroitin Sulfate N-Acetylgalactosaminyltransferase 1	NM_018371	2.38
F2RL1	Coagulation Factor II (Thrombin) Receptor-Like 1	BE965369	2.41
F2RL1	Coagulation Factor II (Thrombin) Receptor-Like 1	NM_005242	2.86
NF2	Neurofibromin 2 (Merlin)	NM_016418	2.49
NF2	Neurofibromin 2 (Merlin)	AF123570	2.18
NF2	Neurofibromin 2 (Merlin)	AF122828	2.33
NF2	Neurofibromin 2 (Merlin)	S73854	1.78
NF2	Neurofibromin 2 (Merlin)	BE313317	1.30
NF2	Neurofibromin 2 (Merlin)	AF122827	1.13
ANPEP	Alanyl (Membrane) Aminopeptidase (Aminopeptidase N, Aminopeptidase M, Microsomal Aminopeptidase, CD13, p150)	NM_001150	2.48

Table S2. *Cont.*

Symbol	Description	GenBank	Fold Change (Log_2 Values)
GPR87	G Protein-Coupled Receptor 87	NM_023915	2.54
F2R	Coagulation Factor II (Thrombin) Receptor	NM_001992	2.61
COL2A1	Collagen, Type II, Alpha 1	X06268	2.63
COL2A1	Collagen, Type II, Alpha 1	X16468	2.74
COL8A1	Collagen, Type VIII, Alpha 1	AL359062	2.63
CXCL6	Chemokine (C-X-C motif) Ligand 6 (Granulocyte Chemotactic Protein 2)	NM_002993	2.63
C5	Complement Component 5	NM_001735	2.69
RAMP1	Receptor (G protein-Coupled) Activity Modifying Protein 1	NM_005855	2.69
COL13A1	Collagen, Type XIII, Alpha 1	M33653	2.75
COL13A1	Collagen, Type XIII, Alpha 1	M59217	1.92
COL13A1	Collagen, Type XIII, Alpha 1	NM_005203	1.42
GRPR	Gastrin-Releasing Peptide Receptor	NM_005314	2.82
COL3A1	Collagen, Type III, Alpha 1 (Ehlers-Danlos Syndrome Type IV, Autosomal Dominant)	AU144167	2.89
COL3A1	Collagen, Type III, Alpha 1 (Ehlers-Danlos Syndrome Type IV, Autosomal Dominant)	AI813758	2.53
ITGBL1	Integrin, Beta-Like 1 (with EGF-Like Repeat Domains)	AI753143	2.99
ITGBL1	Integrin, Beta-Like 1 (with EGF-Like Repeat Domains)	AL359052	2.67
ITGBL1	Integrin, Beta-Like 1 (with EGF-Like Repeat Domains)	AK026784	2.70
ITGBL1	Integrin, Beta-Like 1 (with EGF-Like Repeat Domains)	NM_004791	1.82
NEDD9	Neural Precursor Cell Expressed, Developmentally Down-Regulated 9	AL136139	3.00
NEDD9	Neural Precursor Cell Expressed, Developmentally Down-Regulated 9	U64317	1.58
SOX9	SRY (Sex Determining Region Y)-Box 9 (Campomelic Dysplasia, Autosomal Sex-Reversal)	AI382146	3.00

Table S2. *Cont.*

Symbol	Description	GenBank	Fold Change (Log2 Values)
SOX9	SRY (Sex Determining Region Y)-Box 9 (Campomelic Dysplasia, Autosomal Sex-Reversal)	NM_000346	2.97
GNA14	Guanine Nucleotide Binding Protein (G protein), Alpha 14	NM_004297	3.15
PLA2G7	Phospholipase A2, Group VII (Platelet-Activating Factor Acetylhydrolase, Plasma)	NM_005084	3.20
LGR5	Leucine-Rich Repeat-Containing G Protein-Coupled Receptor 5	AL524520	3.23
LGR5	Leucine-Rich Repeat-Containing G Protein-Coupled Receptor 5	AF062006	1.75
PPAP2B	Phosphatidic Acid Phosphatase Type 2B	AV725664	3.29
PPAP2B	Phosphatidic Acid Phosphatase Type 2B	AB000889	3.16
PPAP2B	Phosphatidic Acid Phosphatase Type 2B	AA628586	2.89
CTGF	Connective Tissue Growth Factor	M92934	4.25
CLDN11	Claudin 11 (Oligodendrocyte Transmembrane Protein)	AW264204	4.49

Table S3. David analysis of GO categories modulated by CCG-1423. Genes highlighted in red represent highly significant downregulated genes modulated by CCG-1423.

**GENE ONTOLOGY (DAVID Bioinformatics Resource http://david.abcc.ncifcrf.gov/home.jsp)**				
**A. UPREGULATED GENES 24h Post-CCG1423 Exposure**				
**Biologic Process**	**# of Genes**	**Fold Enrichment**	***p* Value**	**Benjamini**
GO:0010033~response to organic substance	69	2.20	9.93E-10	2.86E-06
GO:0043067~regulation of programmed cell death	73	2.06	4.51E-09	6.50E-06
GO:0010941~regulation of cell death	73	2.06	5.31E-09	5.10E-06
GO:0042981~regulation of apoptosis	72	2.06	7.00E-09	5.04E-06
GO:0034976~response to endoplasmic reticulum stress	12	8.11	1.09E-07	6.31E-05
GO:0034620~cellular response to unfolded protein	9	9.84	1.50E-06	7.18E-04
GO:0030968~endoplasmic reticulum unfolded protein response	9	9.84	1.50E-06	7.18E-04
GO:0006984~ER-nuclear signaling pathway	11	7.22	1.53E-06	6.29E-04
GO:0009719~response to endogenous stimulus	40	2.27	2.70E-06	9.70E-04

Table S3. *Cont.*

**GENE ONTOLOGY (DAVID Bioinformatics Resource http://david.abcc.ncifcrf.gov/home.jsp)**				
**A. UPREGULATED GENES 24h Post-CCG1423 Exposure**				
**Biologic Process**	**# of Genes**	**Fold Enrichment**	***p* Value**	**Benjamini**
GO:0008285~negative regulation of cell proliferation	37	2.35	2.95E-06	9.45E-04
GO:0031667~response to nutrient levels	24	2.80	1.55E-05	4.44E-03
GO:0032103~positive regulation of response to external stimulus	13	4.67	1.68E-05	4.39E-03
GO:0032101~regulation of response to external stimulus	21	3.03	1.85E-05	4.44E-03
GO:0009991~response to extracellular stimulus	25	2.61	3.16E-05	6.97E-03
GO:0070482~response to oxygen levels	19	3.09	3.94E-05	8.07E-03
GO:0006986~response to unfolded protein	13	4.21	4.96E-05	9.49E-03
GO:0042127~regulation of cell proliferation	59	1.72	5.13E-05	9.19E-03
GO:0051789~response to protein stimulus	16	3.43	5.79E-05	9.77E-03
GO:0009725~response to hormone stimulus	34	2.13	6.27E-05	9.98E-03
GO:0001666~response to hypoxia	18	3.09	6.94E-05	1.05E-02
GO:0048146~positive regulation of fibroblast proliferation	8	7.07	9.03E-05	1.29E-02
GO:0048145~regulation of fibroblast proliferation	9	5.91	1.01E-04	1.37E-02
GO:0051270~regulation of cell motion	22	2.62	1.01E-04	1.31E-02
GO:0007584~response to nutrient	18	2.95	1.20E-04	1.50E-02
GO:0048660~regulation of smooth muscle cell proliferation	10	4.99	1.36E-04	1.62E-02
GO:0030334~regulation of cell migration	20	2.72	1.39E-04	1.59E-02
GO:0055114~oxidation reduction	49	1.76	1.49E-04	1.64E-02
GO:0043066~negative regulation of apoptosis	32	2.08	1.69E-04	1.78E-02
GO:0001568~blood vessel development	25	2.34	1.73E-04	1.77E-02
GO:0043069~negative regulation of programmed cell death	32	2.05	2.18E-04	2.15E-02
GO:0060548~negative regulation of cell death	32	2.04	2.27E-04	2.16E-02
GO:0050729~positive regulation of inflammatory response	8	6.12	2.40E-04	2.21E-02
GO:0001944~vasculature development	25	2.29	2.50E-04	2.23E-02
GO:0040012~regulation of locomotion	21	2.51	2.64E-04	2.28E-02
GO:0043065~positive regulation of apoptosis	36	1.92	2.74E-04	2.29E-02
GO:0031349~positive regulation of defense response	12	3.78	2.92E-04	2.38E-02

Table S3. *Cont.*

GENE ONTOLOGY (DAVID Bioinformatics Resource http://david.abcc.ncifcrf.gov/home.jsp)				
**A. UPREGULATED GENES 24h Post-CCG1423 Exposure**				
**Biologic Process**	**# of Genes**	**Fold Enrichment**	***p* Value**	**Benjamini**
GO:0043068~positive regulation of programmed cell death	36	1.91	3.09E-04	2.44E-02
GO:0051272~positive regulation of cell motion	14	3.28	3.12E-04	2.40E-02
GO:0040017~positive regulation of locomotion	14	3.28	3.12E-04	2.40E-02
GO:0002237~response to molecule of bacterial origin	13	3.47	3.28E-04	2.45E-02
GO:0010942~positive regulation of cell death	36	1.90	3.37E-04	2.46E-02
GO:0008219~cell death	52	1.66	3.73E-04	2.65E-02
GO:0008610~lipid biosynthetic process	29	2.06	4.01E-04	2.78E-02
GO:0016053~organic acid biosynthetic process	18	2.67	4.12E-04	2.79E-02
GO:0046394~carboxylic acid biosynthetic process	18	2.67	4.12E-04	2.79E-02
GO:0050727~regulation of inflammatory response	12	3.63	4.18E-04	2.76E-02
GO:0016265~death	52	1.65	4.29E-04	2.77E-02
GO:0030335~positive regulation of cell migration	13	3.35	4.52E-04	2.85E-02
GO:0009611~response to wounding	41	1.78	4.80E-04	2.96E-02
GO:0033273~response to vitamin	11	3.83	5.22E-04	3.15E-02
GO:0016044~membrane organization	32	1.93	6.03E-04	3.55E-02
GO:0016126~sterol biosynthetic process	8	5.25	6.57E-04	3.79E-02
GO:0006916~anti-apoptosis	21	2.34	6.60E-04	3.73E-02
GO:0009069~serine family amino acid metabolic process	7	6.18	7.18E-04	3.98E-02
**Molecular Function**	**Count**	**Fold Enrichment**	***p* Value**	**Benjamini**
GO:0046983~protein dimerization activity	46	1.98	1.55E-05	1.36E-02
GO:0048037~cofactor binding	27	2.53	2.46E-05	1.08E-02
**Cellular Compartment**	**Count**	**Fold Enrichment**	***p* Value**	**Benjamini**
GO:0005783~endoplasmic reticulum	76	1.89	7.66E-08	2.83E-05
GO:0005829~cytosol	88	1.58	1.30E-05	2.40E-03
GO:0044432~endoplasmic reticulum part	32	2.20	5.58E-05	6.85E-03
GO:0005788~endoplasmic reticulum lumen	13	3.88	1.12E-04	1.03E-02
GO:0000267~cell fraction	69	1.52	4.14E-04	3.01E-02
GO:0031988~membrane-bounded vesicle	41	1.72	8.32E-04	4.99E-02

Table S3. *Cont.*

GENE ONTOLOGY (DAVID Bioinformatics Resource http://david.abcc.ncifcrf.gov/home.jsp)				
**B. DOWNREGULATED GENES 24h Post-CCG1423 Exposure**				
**Biologic Process**	**Count**	**Fold Enrichment**	***p* Value**	**Benjamini**
GO:0007049~cell cycle	189	3.30	2.01E-52	6.60E-49
GO:0000279~M phase	113	4.65	2.51E-46	4.13E-43
GO:0022403~cell cycle phase	127	4.15	4.31E-46	4.73E-43
GO:0022402~cell cycle process	141	3.38	8.25E-40	6.79E-37
GO:0000087~M phase of mitotic cell cycle	84	5.08	1.43E-37	9.42E-35
GO:0051301~cell division	96	4.41	5.00E-37	2.74E-34
GO:0000280~nuclear division	82	5.05	1.91E-36	8.97E-34
GO:0007067~mitosis	82	5.05	1.91E-36	8.97E-34
GO:0048285~organelle fission	82	4.85	5.57E-35	2.29E-32
GO:0000278~mitotic cell cycle	105	3.84	1.21E-34	4.42E-32
GO:0006259~DNA metabolic process	122	3.26	9.36E-33	3.08E-30
GO:0006260~DNA replication	69	4.92	7.27E-30	2.17E-27
GO:0007059~chromosome segregation	38	6.35	4.22E-21	1.16E-18
GO:0006281~DNA repair	70	3.34	2.04E-19	5.17E-17
GO:0006974~response to DNA damage stimulus	80	2.90	3.08E-18	7.24E-16
GO:0051726~regulation of cell cycle	67	2.74	5.76E-14	1.26E-11
GO:0033554~cellular response to stress	93	2.23	2.70E-13	5.56E-11
GO:0000070~mitotic sister chromatid segregation	20	7.52	6.74E-13	1.31E-10
GO:0007051~spindle organization	22	6.62	9.38E-13	1.72E-10
GO:0000819~sister chromatid segregation	20	7.32	1.29E-12	2.24E-10
GO:0007017~microtubule-based process	54	2.89	2.50E-12	4.11E-10
GO:0006261~DNA-dependent DNA replication	23	5.37	4.28E-11	6.71E-09
GO:0000226~microtubule cytoskeleton organization	37	3.41	8.71E-11	1.30E-08
GO:0051276~chromosome organization	77	2.15	2.16E-10	3.09E-08
GO:0008283~cell proliferation	71	2.21	3.91E-10	5.36E-08
GO:0000075~cell cycle checkpoint	27	4.02	1.04E-09	1.37E-07
GO:0051321~meiotic cell cycle	28	3.79	1.98E-09	2.51E-07
GO:0007126~meiosis	27	3.73	5.92E-09	7.21E-07

Table S3. *Cont.*

GENE ONTOLOGY (DAVID Bioinformatics Resource http://david.abcc.ncifcrf.gov/home.jsp)				
**B. DOWNREGULATED GENES 24h Post-CCG1423 Exposure**				
**Biologic Process**	**Count**	**Fold Enrichment**	***p* Value**	**Benjamini**
GO:0051327~M phase of meiotic cell cycle	27	3.73	5.92E-09	7.21E-07
GO:0010564~regulation of cell cycle process	28	3.33	4.21E-08	4.95E-06
GO:0006297~nucleotide-excision repair, DNA gap filling	11	8.76	5.51E-08	6.25E-06
GO:0006310~DNA recombination	26	3.35	1.19E-07	1.30E-05
GO:0043933~macromolecular complex subunit organization	90	1.72	4.13E-07	4.39E-05
GO:0006270~DNA replication initiation	10	8.46	4.45E-07	4.58E-05
GO:0007346~regulation of mitotic cell cycle	31	2.76	5.68E-07	5.66E-05
GO:0065003~macromolecular complex assembly	84	1.71	1.24E-06	1.20E-04
GO:0006461~protein complex assembly	67	1.80	3.41E-06	3.21E-04
GO:0070271~protein complex biogenesis	67	1.80	3.41E-06	3.21E-04
GO:0051329~interphase of mitotic cell cycle	23	3.02	4.70E-06	4.30E-04
GO:0006302~double-strand break repair	17	3.71	7.35E-06	6.53E-04
GO:0051325~interphase	23	2.94	7.69E-06	6.66E-04
GO:0007010~cytoskeleton organization	59	1.83	7.83E-06	6.60E-04
GO:0034621~cellular macromolecular complex subunit organization	51	1.93	8.06E-06	6.63E-04
GO:0006323~DNA packaging	24	2.78	1.24E-05	9.96E-04
GO:0007076~mitotic chromosome condensation	8	8.33	1.35E-05	1.05E-03
GO:0051052~regulation of DNA metabolic process	23	2.73	2.58E-05	1.97E-03
GO:0006289~nucleotide-excision repair	15	3.69	3.21E-05	2.40E-03
GO:0034622~cellular macromolecular complex assembly	45	1.92	3.77E-05	2.75E-03
GO:0030261~chromosome condensation	10	5.42	4.33E-05	3.09E-03
GO:0007093~mitotic cell cycle checkpoint	13	4.09	4.38E-05	3.06E-03
GO:0050000~chromosome localization	8	7.22	4.43E-05	3.03E-03
GO:0007052~mitotic spindle organization	8	7.22	4.43E-05	3.03E-03
GO:0051303~establishment of chromosome localization	8	7.22	4.43E-05	3.03E-03
GO:0048015~phosphoinositide-mediated signaling	19	2.92	6.20E-05	4.16E-03
GO:0007018~microtubule-based movement	22	2.64	6.97E-05	4.58E-03
GO:0006284~base-excision repair	10	5.02	8.67E-05	5.58E-03

Table S3. *Cont.*

GENE ONTOLOGY (DAVID Bioinformatics Resource http://david.abcc.ncifcrf.gov/home.jsp)				
**B. DOWNREGULATED GENES 24h Post-CCG1423 Exposure**				
**Biologic Process**	**Count**	**Fold Enrichment**	***p* Value**	**Benjamini**
GO:0006221~pyrimidine nucleotide biosynthetic process	9	5.54	1.07E-04	6.76E-03
GO:0006271~DNA strand elongation during DNA replication	5	13.54	1.39E-04	8.57E-03
GO:0032392~DNA geometric change	8	6.02	1.80E-04	1.09E-02
GO:0032508~DNA duplex unwinding	8	6.02	1.80E-04	1.09E-02
GO:0034404~nucleobase, nucleoside and nucleotide biosynthetic process	30	2.10	1.87E-04	1.11E-02
GO:0034654~nucleobase, nucleoside, nucleotide and nucleic acid biosynthetic process	30	2.10	1.87E-04	1.11E-02
GO:0006220~pyrimidine nucleotide metabolic process	11	4.14	1.97E-04	1.15E-02
GO:0000724~double-strand break repair via homologous recombination	8	5.70	2.67E-04	1.53E-02
GO:0000725~recombinational repair	8	5.70	2.67E-04	1.53E-02
GO:0006268~DNA unwinding during replication	7	6.77	2.85E-04	1.60E-02
GO:0033043~regulation of organelle organization	32	2.00	2.88E-04	1.59E-02
GO:0031570~DNA integrity checkpoint	13	3.39	3.18E-04	1.73E-02
GO:0009314~response to radiation	30	2.03	3.46E-04	1.85E-02
GO:0022616~DNA strand elongation	5	11.28	3.91E-04	2.06E-02
GO:0051293~establishment of spindle localization	6	8.12	3.98E-04	2.06E-02
GO:0051653~spindle localization	6	8.12	3.98E-04	2.06E-02
GO:0000079~regulation of cyclin-dependent protein kinase activity	13	3.26	4.60E-04	2.34E-02
GO:0009262~deoxyribonucleotide metabolic process	9	4.51	5.34E-04	2.67E-02
GO:0009165~nucleotide biosynthetic process	28	2.04	5.36E-04	2.64E-02
GO:0051172~negative regulation of nitrogen compound metabolic process	60	1.57	5.55E-04	2.69E-02
GO:0000910~cytokinesis	11	3.63	6.21E-04	2.96E-02
GO:0007088~regulation of mitosis	13	3.14	6.54E-04	3.07E-02
GO:0051783~regulation of nuclear division	13	3.14	6.54E-04	3.07E-02
GO:0045934~negative regulation of nucleobase, nucleoside, nucleotide and nucleic acid metabolic process	59	1.56	6.71E-04	3.11E-02
GO:0032886~regulation of microtubule-based process	12	3.32	7.19E-04	3.28E-02
GO:0070507~regulation of microtubule cytoskeleton organization	11	3.55	7.62E-04	3.43E-02
GO:0043623~cellular protein complex assembly	25	2.09	7.96E-04	3.53E-02

Table S3. *Cont.*

GENE ONTOLOGY (DAVID Bioinformatics Resource http://david.abcc.ncifcrf.gov/home.jsp)				
**B. DOWNREGULATED GENES 24h Post-CCG1423 Exposure**				
**Biologic Process**	**Count**	**Fold Enrichment**	***p* Value**	**Benjamini**
GO:0051323~metaphase	5	9.67	8.60E-04	3.75E-02
GO:0009792~embryonic development ending in birth or egg hatching	42	1.70	8.79E-04	3.79E-02
GO:0051640~organelle localization	17	2.50	0.00104692	4.43E-02
GO:0031577~spindle checkpoint	6	6.77	0.00110485	4.61E-02
**Molecular Function**	**Count**	**Fold Enrichment**	***p* Value**	**Benjamini**
GO:0032559~adenyl ribonucleotide binding	177	1.68	9.33E-13	9.96E-10
GO:0005524~ATP binding	175	1.68	1.10E-12	5.90E-10
GO:0030554~adenyl nucleotide binding	181	1.63	6.73E-12	2.39E-09
GO:0001882~nucleoside binding	183	1.61	1.30E-11	3.47E-09
GO:0001883~purine nucleoside binding	181	1.61	2.46E-11	5.26E-09
GO:0000166~nucleotide binding	233	1.48	8.29E-11	1.48E-08
GO:0008094~DNA-dependent ATPase activity	21	5.24	6.97E-10	1.06E-07
GO:0032553~ribonucleotide binding	194	1.50	1.41E-09	1.88E-07
GO:0032555~purine ribonucleotide binding	194	1.50	1.41E-09	1.88E-07
GO:0017076~purine nucleotide binding	198	1.47	6.07E-09	7.20E-07
GO:0003678~DNA helicase activity	16	5.69	3.40E-08	3.63E-06
GO:0042802~identical protein binding	78	1.73	1.92E-06	1.86E-04
GO:0004003~ATP-dependent DNA helicase activity	11	6.26	3.44E-06	3.06E-04
GO:0003697~single-stranded DNA binding	16	4.14	3.70E-06	3.04E-04
GO:0004386~helicase activity	27	2.74	4.18E-06	3.19E-04
GO:0043566~structure-specific DNA binding	25	2.45	6.92E-05	0.00491228
GO:0003777~microtubule motor activity	17	3.14	7.22E-05	0.00480558
GO:0003682~chromatin binding	25	2.37	1.20E-04	0.00749106
GO:0016887~ATPase activity	43	1.83	1.63E-04	0.0096445
GO:0042623~ATPase activity, coupled	36	1.88	3.60E-04	0.02004258
GO:0003774~motor activity	23	2.30	3.61E-04	0.01910098
GO:0046983~protein dimerization activity	60	1.57	4.86E-04	0.02439864
GO:0003684~damaged DNA binding	12	3.41	5.71E-04	0.02734409
GO:0005160~transforming growth factor beta receptor binding	7	5.86	7.46E-04	0.03406755
GO:0016538~cyclin-dependent protein kinase regulator activity	7	5.86	7.46E-04	0.03406755
GO:0004672~protein kinase activity	64	1.50	1.05E-03	0.045767

Table S3. *Cont.*

GENE ONTOLOGY (DAVID Bioinformatics Resource http://david.abcc.ncifcrf.gov/home.jsp)				
**B. DOWNREGULATED GENES 24h Post-CCG1423 Exposure**				
**Cellular Compartment**	**Count**	**Fold Enrichment**	***p* Value**	**Benjamini**
GO:0044427~chromosomal part	100	3.63	9.31E-31	5.14E-28
GO:0005694~chromosome	110	3.35	1.45E-30	3.99E-28
GO:0000775~chromosome, centromeric region	53	5.99	1.11E-27	2.04E-25
GO:0000793~condensed chromosome	54	5.87	1.15E-27	1.59E-25
GO:0005819~spindle	51	4.86	7.99E-22	8.82E-20
GO:0000779~condensed chromosome, centromeric region	33	7.01	7.25E-20	6.67E-18
GO:0031981~nuclear lumen	193	1.87	5.48E-19	4.32E-17
GO:0005654~nucleoplasm	137	2.18	6.44E-19	4.45E-17
GO:0043228~non-membrane-bounded organelle	294	1.59	7.35E-19	4.51E-17
GO:0043232~intracellular non-membrane-bounded organelle	294	1.59	7.35E-19	4.51E-17
GO:0000777~condensed chromosome kinetochore	30	7.25	1.34E-18	7.40E-17
GO:0031974~membrane-enclosed lumen	228	1.72	2.86E-18	1.44E-16
GO:0043233~organelle lumen	223	1.72	1.06E-17	4.87E-16
GO:0000776~kinetochore	33	6.01	2.17E-17	9.21E-16
GO:0070013~intracellular organelle lumen	215	1.69	4.62E-16	1.75E-14
GO:0015630~microtubule cytoskeleton	94	2.40	1.63E-15	6.13E-14
GO:0000228~nuclear chromosome	38	3.29	1.51E-10	5.21E-09
GO:0005657~replication fork	16	7.01	9.96E-10	3.23E-08
GO:0000922~spindle pole	16	6.60	2.85E-09	8.75E-08
GO:0005874~microtubule	49	2.51	4.74E-09	1.38E-07
GO:0005876~spindle microtubule	14	6.77	2.61E-08	7.20E-07
GO:0044430~cytoskeletal part	112	1.65	9.80E-08	2.58E-06
GO:0005856~cytoskeleton	147	1.49	3.76E-07	9.44E-06
GO:0005813~centrosome	38	2.38	1.24E-06	2.97E-05
GO:0044454~nuclear chromosome part	26	2.99	1.28E-06	2.93E-05
GO:0005815~microtubule organizing center	41	2.27	1.47E-06	3.24E-05

Table S3. *Cont.*

**GENE ONTOLOGY (DAVID Bioinformatics Resource http://david.abcc.ncifcrf.gov/home.jsp)**				
**B. DOWNREGULATED GENES 24h Post-CCG1423 Exposure**				
**Cellular Compartment**	**Count**	**Fold Enrichment**	***p* Value**	**Benjamini**
GO:0000785~chromatin	34	2.38	4.73E-06	1.00E-04
GO:0000940~outer kinetochore of condensed chromosome	7	10.90	8.99E-06	1.84E-04
GO:0000796~condensin complex	6	14.02	1.03E-05	2.03E-04
GO:0005730~nucleolus	77	1.55	1.19E-04	0.00225319
GO:0000794~condensed nuclear chromosome	13	3.72	1.26E-04	0.00230761
GO:0005635~nuclear envelope	31	2.12	1.27E-04	0.00226633
GO:0030496~midbody	8	6.23	1.45E-04	0.00249115
GO:0043601~nuclear replisome	7	7.01	2.35E-04	0.00393046
GO:0030894~replisome	7	7.01	2.35E-04	0.00393046
GO:0005658~alpha DNA polymerase:primase complex	5	11.68	3.42E-04	0.00554272
GO:0005663~DNA replication factor C complex	5	11.68	3.42E-04	0.00554272
GO:0043596~nuclear replication fork	7	6.54	3.69E-04	0.00580038
GO:0005829~cytosol	127	1.34	4.30E-04	0.00657576
GO:0000307~cyclin-dependent protein kinase holoenzyme complex	6	7.64	5.85E-04	0.00868525
GO:0005581~collagen	10	4.00	5.94E-04	0.00860088
GO:0031967~organelle envelope	67	1.51	6.19E-04	0.00871928
GO:0031975~envelope	67	1.51	6.71E-04	0.00921911
GO:0044451~nucleoplasm part	60	1.52	1.22E-03	0.01629899
GO:0031262~Ndc80 complex	4	14.02	1.37E-03	0.01781538
GO:0000792~heterochromatin	10	3.42	2.01E-03	0.02544686
GO:0005667~transcription factor complex	28	1.87	2.03E-03	0.0251575
GO:0005681~spliceosome	20	2.12	2.53E-03	0.03063395
GO:0034399~nuclear periphery	12	2.76	3.52E-03	0.04146841

Table S4. GSEA MSigDB datasets modulated by CCG-1423. Gene sets that are modulated by CCG-1423 and involve the E2F transcriptional program are highlighted in blue, gene sets involved in melanoma are highlighted in red, and gene sets involved in cancer therapeutics are highlighted in yellow.

	**Gene Sets Significantly Related to Those Modulated by CCG-1423 but not DRB**					
**LatB**	**ID**	**Log(Fold Change)**	***T***	***p*. Value**	**adj.P.Val**	***B***
Y	GRAHAM_NORMAL_QUIESCENT_VS_NORMAL_DIVIDING_DN	−27.14	−20.1409	2.03E-09	2.32E-06	8.908373
Y	KANG_DOXORUBICIN_RESISTANCE_UP	−26.3657	−18.0326	5.96E-09	2.32E-06	8.453042
Y	LEE_EARLY_T_LYMPHOCYTE_UP	−24.6309	−18.0169	6.01E-09	2.32E-06	8.449227
Y	CHANG_CYCLING_GENES	−18.8982	−15.1802	3.15E-08	4.35E-06	7.628781
Y	FRASOR_RESPONSE_TO_SERM_OR_FULVESTRANT_DN	−16.1085	−14.811	3.99E-08	4.35E-06	7.499588
Y	ODONNELL_TFRC_TARGETS_DN	−21.662	−14.8342	3.93E-08	4.35E-06	7.5079
Y	REN_BOUND_BY_E2F	−16.1562	−15.4961	2.58E-08	4.35E-06	7.734719
Y	ROSTY_CERVICAL_CANCER_PROLIFERATION_CLUSTER	−34.6161	−15.6642	2.33E-08	4.35E-06	7.789425
Y	SOTIRIOU_BREAST_CANCER_GRADE_1_VS_3_UP	−31.3439	−15.7063	2.27E-08	4.35E-06	7.802955
Y	WHITEFORD_PEDIATRIC_CANCER_MARKERS	−24.705	−15.0774	3.36E-08	4.35E-06	7.593405
Y	ZHAN_MULTIPLE_MYELOMA_PR_UP	−24.2928	−15.2314	3.05E-08	4.35E-06	7.646246
Y	ODONNELL_TARGETS_OF_MYC_AND_TFRC_DN	−15.1333	−14.3945	5.24E-08	4.75E-06	7.34645
Y	REACTOME_E2F_TRANSCRIPTIONAL_TARGETS_AT_G1_S	−9.17733	−14.3735	5.31E-08	4.75E-06	7.338506
	PODAR_RESPONSE_TO_ADAPHOSTIN_UP	19.29053	14.40544	5.20E-08	4.75E-06	7.350588
Y	SONG_TARGETS_OF_IE86_CMV_PROTEIN	−14.584	−14.1263	6.27E-08	5.35E-06	7.243482
	TIEN_INTESTINE_PROBIOTICS_24HR_DN	16.48571	14.06688	6.53E-08	5.35E-06	7.220188
Y	WINNEPENNINCKX_MELANOMA_METASTASIS_UP	−25.2913	−13.7934	7.88E-08	5.95E-06	7.110667
	FURUKAWA_DUSP6_TARGETS_PCI35_DN	−18.0625	−13.5977	9.03E-08	6.33E-06	7.029855
	ZHAN_MULTIPLE_MYELOMA_CD1_VS_CD2_UP	9.240549	13.59875	9.02E-08	6.33E-06	7.030311
Y	KAUFFMANN_DNA_REPLICATION_GENES	−10.3129	−13.0806	1.30E-07	7.78E-06	6.806271
Y	MOLENAAR_TARGETS_OF_CCND1_AND_CDK4_DN	−17.8292	−13.15	1.24E-07	7.78E-06	6.83715
Y	REACTOME_DNA_STRAND_ELONGATION	−12.2779	−13.0779	1.31E-07	7.78E-06	6.805058
	BLUM_RESPONSE_TO_SALIRASIB_UP	19.21202	13.082	1.30E-07	7.78E-06	6.806894
	TANG_SENESCENCE_TP53_TARGETS_DN	−15.4327	−12.9833	1.40E-07	7.86E-06	6.762481
Y	BENPORATH_PROLIFERATION	−19.7266	−12.8858	1.50E-07	8.21E-06	6.718053
	FARMER_BREAST_CANCER_CLUSTER_2	−19.7857	−12.8206	1.58E-07	8.25E-06	6.687978
	SHEPARD_BMYB_MORPHOLINO_DN	−10.3705	−12.8051	1.60E-07	8.25E-06	6.680798
Y	WONG_EMBRYONIC_STEM_CELL_CORE	−23.3314	−12.7381	1.68E-07	8.28E-06	6.649633

Table S4. *Cont.*

LatB	ID	Log(Fold Change)	*T*	*p*. Value	adj.P.Val	*B*
	**Gene Sets Significantly Related to Those Modulated by CCG-1423 but not DRB**					
**LatB**	**ID**	**Log(Fold Change)**	***T***	***p*. Value**	**adj.P.Val**	***B***
Y	KAUFFMANN_MELANOMA_RELAPSE_UP	−14.0755	−12.6697	1.77E-07	8.46E-06	6.617482
Y	REACTOME_E2F_MEDIATED_REGULATION_OF_DNA_REPLICATION	−10.5686	−12.5269	1.96E-07	9.19E-06	6.54944
Y	PUJANA_BREAST_CANCER_WITH_BRCA1_MUTATED_UP	−14.8755	−12.4202	2.13E-07	9.73E-06	6.497725
Y	REACTOME_G1_S_TRANSITION	−12.6709	−12.3032	2.33E-07	1.01E-05	6.440202
	PACHER_TARGETS_OF_IGF1_AND_IGF2_UP	9.922428	12.24788	2.43E-07	1.02E-05	6.412662
	KORKOLA_TERATOMA	−6.94337	−12.1669	2.59E-07	1.04E-05	6.372005
Y	REACTOME_MITOTIC_M_M_G1_PHASES	−18.1821	−12.0099	2.92E-07	1.15E-05	6.291916
	**Gene Sets Significantly Related to Those Modulated by CCG-1423 as well as by DRB**					
**LatB**	**ID**	**Log (Fold Change)**	***T***	***p* Value**	**adj.P.Val**	***B***
Y	CROONQUIST_NRAS_SIGNALING_DN	−22.2957	−18.8821	3.81E-09	2.32E-06	8.649585
Y	CROONQUIST_IL6_DEPRIVATION_DN	−26.1486	−19.4606	2.84E-09	2.32E-06	8.773017
Y	GRAHAM_CML_DIVIDING_VS_NORMAL_QUIESCENT_UP	−27.7736	−17.7178	7.07E-09	2.32E-06	8.375174
Y	FUJII_YBX1_TARGETS_DN	−23.253	−16.377	1.51E-08	4.25E-06	8.0093
Y	BLUM_RESPONSE_TO_SALIRASIB_DN	−29.9792	−16.0619	1.83E-08	4.35E-06	7.914446
Y	KOBAYASHI_EGFR_SIGNALING_24HR_DN	−35.9388	−14.9246	3.71E-08	4.35E-06	7.539999
	CONCANNON_APOPTOSIS_BY_EPOXOMICIN_DN	−14.1113	−14.8136	3.98E-08	4.35E-06	7.500532
Y	FERREIRA_EWINGS_SARCOMA_UNSTABLE_VS_STABLE_UP	−19.0778	−14.6979	4.29E-08	4.44E-06	7.458802
Y	BOYAULT_LIVER_CANCER_SUBCLASS_G23_UP	−10.7833	−13.8268	7.70E-08	5.95E-06	7.124254
Y	REACTOME_EXTENSION_OF_TELOMERES	−8.45576	−13.1467	1.24E-07	7.78E-06	6.835701
Y	AMUNDSON_GAMMA_RADIATION_RESPONSE	−16.2052	−12.9888	1.39E-07	7.86E-06	6.764964
Y	MISSIAGLIA_REGULATED_BY_METHYLATION_DN	−17.9098	−12.2875	2.36E-07	1.01E-05	6.432396
	RUIZ_TNC_TARGETS_DN	−20.3841	−12.3389	2.27E-07	1.01E-05	6.457824
Y	REACTOME_S_PHASE	−11.8209	−12.2058	2.51E-07	1.03E-05	6.39157
